# Epitope spreading driven by the joint action of CART cells and pharmacological STING stimulation counteracts tumor escape via antigen-loss variants

**DOI:** 10.1136/jitc-2021-003351

**Published:** 2021-11-21

**Authors:** Enrique Conde, Enric Vercher, Marta Soria-Castellano, Jesús Suarez-Olmos, Uxua Mancheño, Edurne Elizalde, M Luis Rodriguez, Javier Glez-Vaz, Noelia Casares, Estefanía Rodríguez-García, Mirja Hommel, Gloria González-Aseguinolaza, Iratxe Uranga-Murillo, Julian Pardo, Gorka Alkorta, Ignacio Melero, Juan Lasarte, Sandra Hervas-Stubbs

**Affiliations:** 1 Programa de Inmunología e Inmunoterapia, Centro de Investigación Médica Aplicada (CIMA), Universidad de Navarra, Pamplona, Spain; 2 Instituto de Investigación Sanitaria de Navarra (IdiSNA), Pamplona, Spain; 3 Programa de Terapia Génica y Regulación de la Expresión Génica, Centro de Investigación Médica Aplicada (CIMA), Universidad de Navarra, Pamplona, Spain; 4 Microbiología Medicina Preventiva y Salud Pública, Universidad de Zaragoza, Zaragoza, Spain; 5 Centro de Investigación Biomédica de Aragón (CIBA), Fundación Instituto de Investigación Sanitaria Aragón (IIS Aragón), Zaragoza, Spain; 6 Fundacion ARAID, Zaragoza, Spain; 7 CIMA LAB Diagnostics, Universidad de Navarra, Pamplona, Spain; 8 Immunología e Immunoterapia, Clínica Universidad de Navarra, Pamplona, Spain; 9 Centro de Investigación Biomédica en Red de Cáncer (CIBERONC), Madrid, Spain; 10 CIBERehd, Instituto de Salud Carlos III, Madrid, Spain

**Keywords:** adaptive immunity, combined modality therapy, immunotherapy, adoptive, receptors, chimeric antigen, tumor escape

## Abstract

**Background:**

Target antigen (Ag) loss has emerged as a major cause of relapse after chimeric antigen receptor T (CART)-cell therapy. We reasoned that the combination of CART cells, with the consequent tumor debulking and release of Ags, together with an immunomodulatory agent, such as the stimulator of interferon gene ligand (STING-L) 2′3′-cyclic GMP-AMP (2′3′-cGAMP), may facilitate the activation of an endogenous response to secondary tumor Ags able to counteract this tumor escape mechanism.

**Methods:**

Mice bearing B16-derived tumors expressing prostate-specific membrane Ag or gp75 were treated systemically with cognate CART cells followed by intratumoral injections of 2′3′-cGAMP. We studied the target Ag inmunoediting by CART cells and the effect of the CART/STING-L combination on the control of STING-L-treated and STING-L-non-treated tumors and on the endogenous antitumor T-cell response. The role of Batf3-dependent dendritic cells (DCs), stimulator of interferon gene (STING) signaling and perforin (Perf)-mediated killing in the efficacy of the combination were analyzed.

**Results:**

Using an immune-competent solid tumor model, we showed that CART cells led to the emergence of tumor cells that lose the target Ag, recreating the cancer immunoediting effect of CART-cell therapy. In this setting, the CART/STING-L combination, but not the monotherapy with CART cells or STING-L, restrained tumor progression and enhanced overall survival, showing abscopal effects on distal STING-L-non-treated tumors. Interestingly, a secondary immune response against non-chimeric antigen receptor-targeted Ags (epitope spreading), as determined by major histocompatibility complex-I-tetramer staining, was fostered and its intensity correlated with the efficacy of the combination. This was consistent with the oligoclonal expansion of host T cells, as revealed by in-depth T-cell receptor repertoire analysis. Moreover, only in the combination group did the activation of endogenous T cells translate into a systemic antitumor response. Importantly, the epitope spreading and the antitumor effects of the combination were fully dependent on host STING signaling and Batf3-dependent DCs, and were partially dependent on Perf release by CART cells. Interestingly, the efficacy of the CART/STING-L treatment also depended on STING signaling in CART cells.

**Conclusions:**

Our data show that 2′3′-cGAMP is a suitable adjuvant to combine with CART-cell therapy, allowing the induction of an endogenous T-cell response that prevents the outgrowth of Ag-loss tumor variants.

## Background

Adoptive T-cell transfer (ACT) of chimeric antigen receptor T (CART) cells represents a revolutionary treatment for hematological malignancies. However, attempts to reproduce this success in solid tumors have to date been disappointing. Barriers to effective CART-cell therapy in solid tumors include limited trafficking to the tumors, low persistence, and impaired functions due to the immunosuppressive and hostile tumor microenvironment (TME). Tumor escape due to antigen (Ag) loss or low Ag expression has become an important resistance mechanism to CART-cell therapy in hematological malignancies^1–3^ and is likely to also emerge as an important barrier to success in solid tumors, which manifest greater heterogeneity in target Ag expression. Indeed, Ag escape and induced adaptive resistance has also been reported in patients with recurrent glioblastoma who were treated with a single dose of CART cells redirected to the epidermal growth factor receptor variant III (EGFRvIII) mutation.^4^ The mechanism responsible for the appearance of Ag-loss variants is the immunoediting effect^5^ of monotarget therapies and the consequent immune selection of cancer cells expressing low or null target Ag levels. Low Ag density may also result from CART-cell trogocytosis.^6^ Target Ag loss or downregulation seriously affects CART cells as the Ag threshold for chimeric antigen receptors (CARs) is higher than that for T-cell receptors (TCRs).^7^

Increasing evidence shows that the phenomenon of epitope spreading is critical to the development of effective antitumor immunity.^8^ Epitope or Ag spreading is a process characterized by the enhancement and diversification of the endogenous T-cell response against antigenic epitopes other than the originally targeted epitope. It usually occurs following initial therapy-mediated tumor destruction, which leads to the release of secondary tumor Ags. Subsequently, these Ags are taken up by professional antigen-presenting cells, such as dendritic cells (DCs), to induce a novo or rescue a pre-existing T-cell response.

There is some evidence that T-cell therapy induces epitope spreading. In a murine CAR model targeting EGFRvIII, mice that were cured of EGFRvIII^+^ tumors later rejected EGFRvIII^−^ tumors when rechallenged, suggesting that epitope spreading can be induced by CART cells.^9^ During CART-cell therapy in patients with gastric cancer, new T-cell clones against tumor neo-Ags have been detected.^10^ In addition, in a clinical trial of a CAR targeting mesothelin, patients who received CART cells also developed an antitumor antibody response.^11^ Epitope spreading has also been reported in tumor infiltrating lymphocyte therapy^12^ and TCR T-cell therapy.^13–16^ However, few studies have addressed the question of whether the Ag spreading derived directly from the action of T cells achieves a meaningful level for therapeutic efficacy. In a recent study, Etxeberria *et al* elegantly demonstrated that only the combination of TCR T cells with inmunostimulating agents, such as interleukin (IL)-12 and anti-CD137 mAb, was able to induce a systemic endogenous tumor-specific T-cell response strong enough to reject local and distal tumors.^15^ Similar findings were observed when ACT was combined with pathogen-based vaccines.^14^

The fact that Ag spreading in T-cell therapy rarely achieves a sufficient level for therapeutic efficacy may be associated with insufficient activation of Batf3-dependent DCs, which are required for the initial cross-priming of CD8 T cells and effector T-cell recruitment into the TME.^17–21^ Gajewski *et al* has shown that activation of the host stimulator of interferon gene (STING) pathway by natural agonist (tumor cell-derived DNA) leads to type I interferon (IFN) production, DC activation, cross-presentation of tumor-associated Ag to CD8 T cells, and T-cell recruitment into the TME.^22^ On the basis of these observations, therapeutic intervention by injecting stimulator of interferon gene ligands (STING-Ls) directly into the tumor has been shown to trigger an antitumor response that induces tumor regression.^23^ Interestingly, Batf3-dependent DCs were critical for the therapeutic effect of STING agonists.^23^

Therefore, we reasoned that the combination of the cytotoxic activity of CART cells, with the consequent tumor debulking and release of Ags, together with the immunostimulatory action of STING-Ls, may facilitate the activation of endogenous T cells specific for secondary tumor Ags. This broadly targeted antitumor immune response may prevent the outgrowth of tumor Ag escape variants and enhance the efficacy of CART therapy.

## Methods

### Mice and cell lines

All mouse strains used in this study are described in the online supplemental Methods. Platinum-E (Cell Biolabs), B16-F10, and B16OVA cells were cultured as described in the online supplemental Methods. Human prostate-specific membrane antigen (hPSMA) expressing B16-F10 (B16-PSMA) tumor cell line was generously provided by Dr F Pastor (CIMA, Spain)^24^ and are described in the online supplemental Methods. Primary mouse T cells were cultured in complete medium (RPMI-1640-glutamax, 10% fetal bovine serum, 100 U/mL penicillin, 100 µg/mL streptomycin, 10 mg/mL gentamicin, 1 mM N-(2-hydroxyethyl)piperazine-N′-(2-ethanesulfonic acid) (HEPES), and 50 mM 2-mercaptoetanhol).

10.1136/jitc-2021-003351.supp1 Supplementary data

### CAR construct design and retroviral transduction of mouse T cells

The anti-human CD19, anti-human/mouse gp75 and anti-human PSMA CARs used in this study are all-mouse proteins. They were designed by fusion of the mouse CD8 protein signal peptide, the scFvs from FMC63 (CD19), TA99 (gp75) or J591 (PSMA) mouse hybridomas, the hinge and transmembrane domain of mouse CD8, the signaling domains of mice CD137 and CD3ζ. Enhanced green fluorescent protein (EGFP) and P2A autocleavage coding sequences were added to the 5′ region of the chimeric gene. The EGFP-P2A-CAR polycistronic gene was cloned into the pRubiC-T2A-CRE retroviral vector^25^ (a gift from B Luikart, Addgene #66692), under the control of the ubiquitin promoter and replacing the Cherry-T2A-cre gene. Retroviral particles were generated as detailed in the online supplemental Methods. Untouched CD8 and CD4 T cells were sorted using the negative selection EasySep Mouse CD8 or CD4 T-cell isolation kits (StemCell), respectively. The CD8 and CD4 T cells, separately, were then activated, cultured, and infected with retroviruses. Cells were harvested on days 4–5 of activation for ACT experiments and for in vitro analyses. Recognition of tumor cells by CART cells was assessed by measuring interferon gamma (IFNγ) production and cytotoxic activity. For more details, see online supplemental Methods.

### Combination therapy and rechallenge experiments

In monolateral tumor models, 8-week-old mice were subcutaneously implanted with 5×10^5^ tumor cells. In bilateral tumor models, mice were subcutaneously implanted with 5×10^5^ and 1.5×10^5^ tumor cells in the right and left flanks, respectively. At the time indicated, mice were treated with an intravenous dose of a mixture (1:1) of CD8 and CD4 CART cells (3 million in total) followed by repetitive intratumoral injections of the STING agonist 2′3′-cyclic GMP-AMP (2′3′-cGAMP) (5 µg/mouse) (Invivogen). In the bilateral tumor model, STING-L was injected intratumorally in the larger right tumor. The 2′3′-cGAMP administration regimen is specified in each figure legend. Mice injected intratumorally with saline were used as control. In certain experiments, STING-KO or perforin (Perf)-KO CART cells were used to compare with wild-type (WT) CART cells. In experiments where the antitumor response was analyzed ex vivo, CD45.1 mice were used for CART-cell generation to distinguish exogenous (CD45.1^+^) from endogenous (CD45.2^+^) T cells. For rechallenge experiments, naïve mice (control) and long-term survivors (greater than 90 days after treatment) of subcutaneous B16-PSMA tumors by combination therapy were inoculated with 5×10^5^ B16-F10 cells on the left flank (contralateral flank for rechallenged mice). Antitumor efficacy was assessed by monitoring tumor size and survival.

### Ex vivo characterization of tumor, CART and endogenous T cells on treatments

For surface staining of the target Ag in in vivo growing tumor cells, tumor-bearing mice (treated as specified in the figure legends) were sacrificed and their tumors homogenized and stained as described in the online supplemental Methods. For ex vivo characterization of CART and endogenous T cells, 2′3′-cGAMP-treated and 2′3′-cGAMP-non-treated tumors, lymph nodes (LNs) (both draining lymph node (DLN) and contralateral lymph node (CLN) (relative to the STING-L-treated tumor)) and blood were processed as described in the online supplemental Methods. Samples were labeled with fluorochrome-labeled mAbs and H-2Kb tetramers loaded with the immune-dominant CD8 T-cell epitopes of B16OVA cells (OVA_257-264_ and MuLV p15E_604-611_, henceforth OVA and M8, respectively) as detailed in the online supplemental Methods. CART cells were identified as GFP^+^CD45.1^+^. The P2A-linked GFP acts as a readout of CAR synthesis and, as compared with detection of scFV using secondary anti-mouse IgG, facilitates the identification of CART cells in multiparametric flow cytometry studies. Cells were acquired in a FACSCanto-II (BD Biosciences) or a CytoFlex (Beckman Coulter) flow cytometer. Data were analyzed with FlowJo software (Tree Star).

### TCR repertoire analysis

In-depth TCR repertoire analysis was conducted in blood and treated tumors using the Oncomine mouse TCR Beta-SR DNA Assay (Thermo Fisher Scientific) as described in the online supplemental methods.

### Statistical analysis

Statistical analyses were performed using GraphPad Prism (V.8.4.0). The statistical tests used have been specified in the legend of each figure.

## Results

### Immune pressure exerted by CART cells drives the selection of tumor cells expressing low target Ag levels

**Figure 1:**
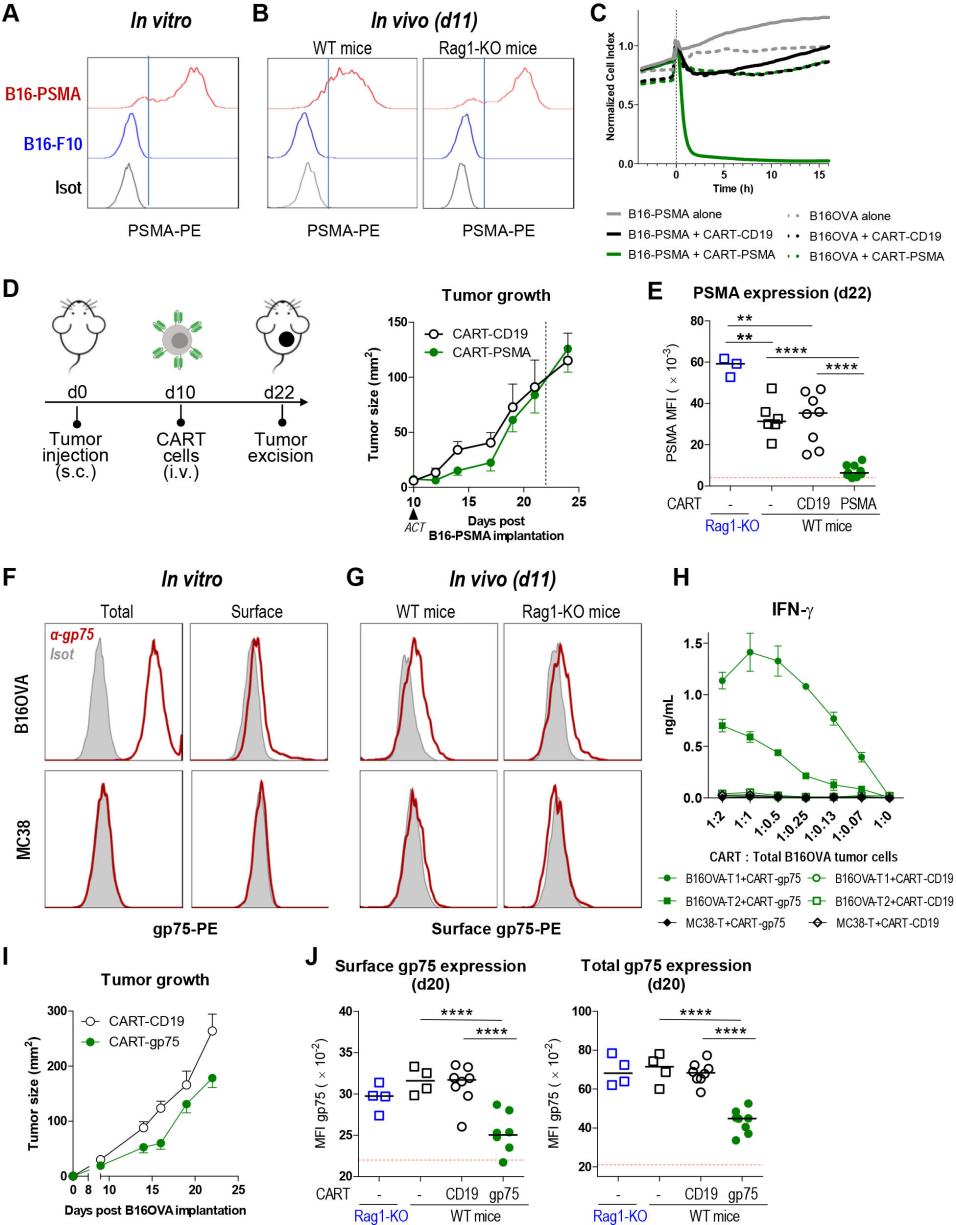
CART cells drive the selection of tumor cells expressing low target Ag levels. (A–E) B16-PSMA/CART-PSMA tumor model. (A) Surface hPSMA expression measured by flow cytometry in in vitro cultured B16-PSMA or B16-F10 cells. (B) Surface expression of hPSMA determined by flow cytometry in tumor cells (CD45^−^FSC^hi^SSC^hi^) from 11-day B16-PSMA or B16-F10 tumor-bearing WT or Rag1-KO mice. (A, B) Staining of B16-PSMA with control isotype is shown as a reference. (C) Real-time cytotoxic activity of CART-PSMA cells. B16-PSMA or B16OVA cells were grown on xCELLigence E-plates. At 24 hours, medium or CART cells (tumor:effector ratio 1:2) were added. Cell index normalized values. (D, E) Ten-day B16-PSMA tumor-bearing B6 mice received an intravenous dose (3×10^6^) of CART-PSMA or CART-CD19 cells. As control, Rag1-KO and B6 (WT) mice bearing B16-PSMA tumors were left untreated to assess PSMA-immunoediting in mice that did not receive CART cells. (D) Experimental model (left) and tumor growth kinetics (right). (E) Mice were sacrificed at day 22 (day 12 of ACT), and surface expression of hPSMA was determined by flow cytometry in tumor cells (CD45^−^FSC^hi^SSC^hi^). (F) Total (surface and intracellular) and surface gp75 expression measured by flow cytometry in in vitro cultured B16OVA and MC38 (negative control) cells. (G) Surface expression of gp75 determined by flow cytometry in tumor cells (CD45^−^FSC^hi^SSC^hi^) from 11-day B16OVA or MC38 tumor-bearing WT or Rag1-KO mice. (F, G) Staining of B16OVA with control isotype is shown as a reference. (H) CD8 CARTs were cocultured with a cell suspension obtained from 10-day implanted B16OVA or MC38 (negative control) tumors (T), and 24 hours later, IFN-γ was measured in the culture supernatant. Cells from homogenized B16OVA tumors from two different mice (T1 and T2) were used. IFN-ɣ levels in the culture supernatant were measured by ELISA. (I) Four-day B16OVA tumor-bearing B6 mice received an intravenous dose (3×10^6^) of CART cells and tumor growth was monitored. (J) Mice treated (as in I) were sacrificed on day 20 (day 16 of ACT) and surface and total gp75 expression was determined in CD45^−^FSC^hi^SSC^hi^ cells from homogenized tumors by flow cytometry. As control, Rag1-KO and B6 (WT) mice bearing B16OVA tumors were left untreated. Red dotted lines depict the average MFI of total (J, right) and surface (E, J; left) staining of tumor cells with the corresponding IgG isotype. N (mice/group)=4 (B, G) and 6 (D, I). (C–E, H–J) One experiment representative of at least two experiments. Data are represented as mean±SD (H), mean±SEM (D, I), mean (C) and median (E, J). Non-parametric Mann-Whitney test, two-tailed (E, J). ****P<0.0001, **P<0,01. ACT, adoptive T-cell transfer; Ag, antigen; CART, chimeric antigen receptor T; IFN-γ, interferon gamma; KO, knockout; MFI, median fluorescence intensity; PSMA, prostate-specific membrane antigen; WT, wild type.

B16-F10 cells were genetically modified to express a hPSMA variant (B16-PSMA) that is not internalized because it lacks the clathrin-binding motif.^24^ Interestingly, cell surface expression of hPSMA was high and quite homogenous in cells cultured in vitro and in B16-PSMA tumors when implanted in Rag1-KO mice, but not in WT mice, where it decreased and became heterogeneous (figure 1A, B). Moreover, B16-PSMA tumors grew more slowly than B16-F10 tumors when implanted in WT mice, while both cell lines exhibited similar growth kinetics in Rag1-KO mice (online supplemental figure S1A). These findings suggested that hPSMA antigenicity in mice had promoted B16-PSMA cell immunoediting in vivo with the consequent increased heterogeneity in hPSMA expression. In order to assess the antitumor efficacy of CART cells targeting this heterogeneously expressed Ag, we designed an all-murine, second-generation CAR specific for hPSMA (online supplemental figure S1B). CAR-PSMA-engineered (CART-PSMA) CD8 T cells expressed the CAR on their surface (online supplemental figure S1C) and efficiently recognized B16-PSMA cells in vitro (figure 1C and online supplemental figure S1D). However, in vivo they only modestly contained tumor progression the first 7 days on ACT (figure 1D). When growing tumors were analyzed 12 days after CART-cell transfer (day 22 of tumor inoculation), tumor cells from WT mice treated with CART-PSMA cells exhibited lower hPSMA surface expression levels than those in the control group (figure 1E). This finding suggested that the immune pressure exerted by CART cells had driven the selection of tumor cells expressing low target Ag levels, which may have favored tumor escape after the initial containment phase.

Similar findings were observed with CART cells targeting a naturally expressed murine Ag, such as gp75 (TYRP1). Although gp75 predominantly localized intracellularly within the melanosomes, it was also exposed at low density on the surface of B16OVA cells (figure 1F), and this surface expression was maintained when implanted in mice (figure 1G). Surface expression of gp75 in WT and Rag1-KO mice was comparable, supporting the notion that mice maintain tolerance to gp75.^26 27^ T cells expressing a CAR against gp75 (online supplemental figure S2A) recognized B16OVA cells isolated from fresh tumors (figure 1H) and slightly delayed tumor growth when injected into 4-day B16OVA-tumor bearing mice (figure 1I). Interestingly, CART-gp75 therapy promoted the selection of tumor cells expressing low gp75 levels, reflected by decreased gp75 intensity after surface and total staining (figure 1J).

### CART cells and intratumoral administration of 2′3′-cGAMP synergize and exert abscopal effects in distal STING-L-non-treated tumors

Given the CART cell-mediated leakage of tumor cells lacking the target Ag and considering the potential of STING agonists to prompt a host’s immune response,^23^ we wondered whether the combination of CART cells with the intratumoral administration of a STING-L might promote an endogenous T-cell response that counteracts tumor escape from CART cells. To test this hypothesis, we selected the STING agonist 2′3′-cGAMP because, unlike other STING-Ls, it does not induce T-cell apoptosis.^28–30^

**Figure 2:**
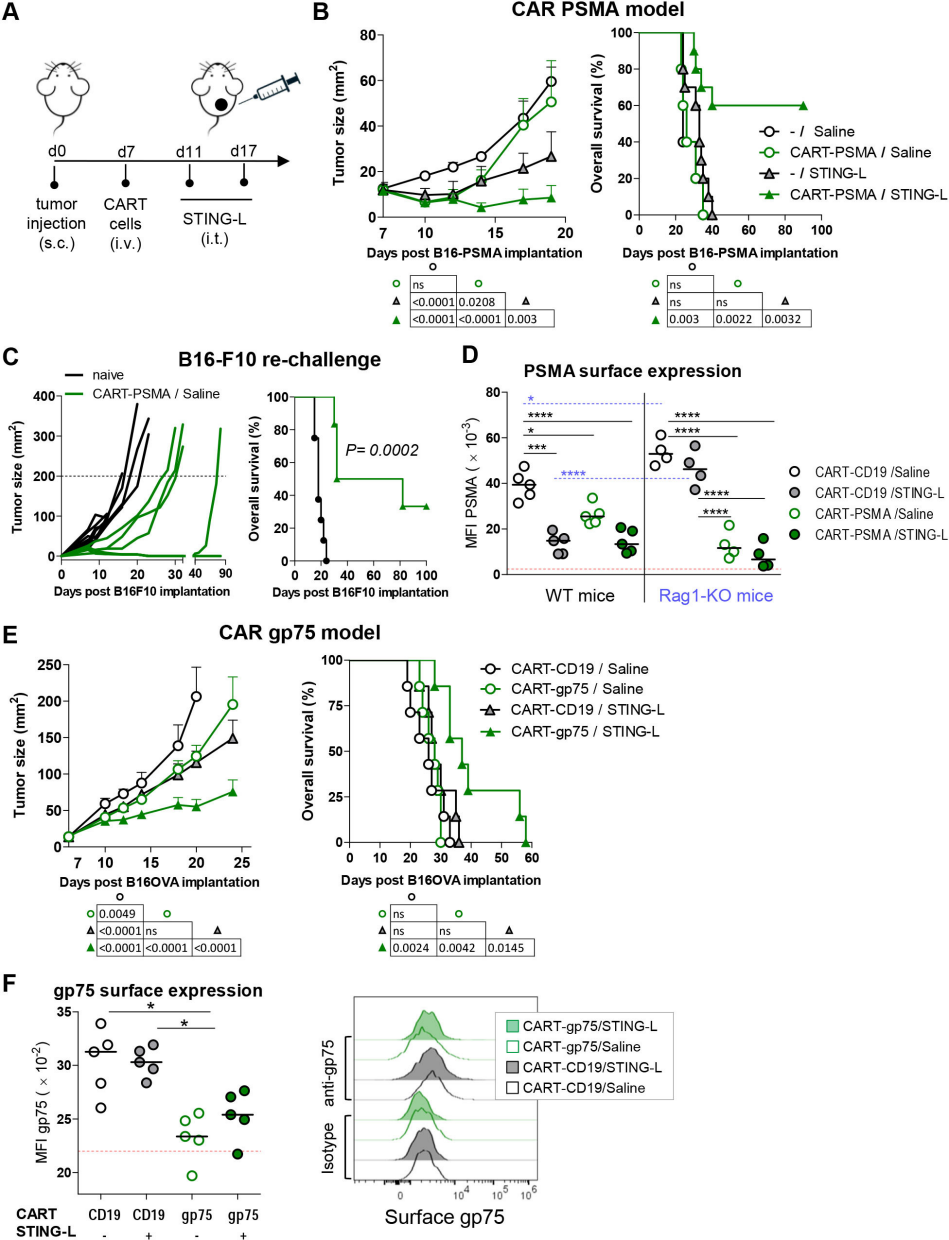
Intratumoral delivery of 2′3′-cGAMP synergizes with CART cells. (A) Experimental model. (B, E) Seven-day B16-PSMA (B) or 6-day B16OVA (E) subcutaneous tumor-bearing B6 mice received an intravenous dose (3×10^6^) of CART-PSMA or CART-gp75 cells. 2′3′-cGAMP (5 µg) or saline was injected intratumorally at days 11 and 17 of tumor implantation. Mice injected with saline (B) or CART-CD19 cells (E) were used as controls. Data represent mean tumor size progression and overall survival of mice. (C) Long-term survivors (more than 90 days after treatment) of subcutaneous B16-PSMA tumors by CART-PSMA/STING-L therapy were rechallenged with 5×10^5^ B16-F10 cells on the contralateral flank and tumor size progression and survival were monitored. Naïve mice were used as controls. (D, F) Mice were treated as those of B and E, respectively. (D) The treatments were also carried out in parallel in Rag1-KO mice. Mice were sacrificed at day 16 or 20 of tumor inoculation corresponding to day 8 or 12 of ACT (D, F, respectively). Surface expression of hPSMA (D) or gp75 (F) was determined by flow cytometry in tumor cells (CD45^−^FSC^hi^SSC^hi^) present in the cell suspension obtained from homogenized tumors. Red dotted lines in box and whiskers graphs depict the average MFI of surface staining of tumor cells with the corresponding IgG isotype. (F) Histograms on the right show MFI of gp75 or control isotype in tumor cells from a representative mouse in each group. N (mice/group)=5 (F), 6 (D), 7 (E), 8 (naïve mice) or 6 (rechallenged mice) (C), and 10 (B). (B–F) One experiment representative of two experiments. Data are represented as mean±SEM (B, D–F). One-way analysis of variance and non-parametric (Kruskal-Wallis) test and Dunns post-test to compare all pairs of groups (D, F). Non-linear regression (curve fit) (B, E; left). Log-rank (Mantel-Cox) test (B, C, E; right). *P<0.05, ***P<0.0005, ****P<0.00005. ACT, adoptive T-cell transfer; CART, chimeric antigen receptor T; MFI, median fluorescence intensity; ns, not significant; PSMA, prostate-specific membrane antigen; STING-L, stimulator of interferon gene ligand.

First, we tested our premise in the B16-PSMA model. Therefore, B16-PSMA tumor-bearing mice were treated with CART cells followed by intratumoral injections of 2′3′-cGAMP (figure 2A). The CART-PSMA/STING-L combination and the therapy with STING-L alone (-/STING-L) or together with unrelated CART cells (CART-CD19) significantly restrained tumor progression (figure 2B and online supplemental figure S1E, F). Interestingly, the combination therapy cured 60%–50% of the mice. Moreover, regions of vitiligo were observed in half of the cured mice (online supplemental figure S1G), suggesting that an endogenous response to tumor-associated Ag, such as melanin, had been induced. Importantly, cured mice showed delayed tumor growth after B16-F10 rechallenge (figure 2C). Strikingly, the rechallenge-resistant mice and the mouse with late-relapse tumor (relapse at day 40) were those exhibiting vitiligo after the combination. Collectively, these data demonstrated a strong synergistic effect of CART cells and 2′3′-cGAMP administration and suggested that the combination promoted the induction of an endogenous T-cell response.

The fact that not all combination-cured mice resisted the rechallenge indicated that either the protection was partial or that the T-cell response had been directed against an Ag not present in B16-F10, such as hPSMA. Interestingly, whereas CART-PSMA/saline treatment shaped PSMA surface expression in both WT and Rag1-KO tumor-bearing mice, the treatment with CART-CD19/STING-L only decreased the expression of hPSMA in WT mice (figure 2D and online supplemental figure S1H), indicating that hPSMA was targeted by both CART-PSMA cells and the 2′3′-cGAMP-induced immune response. Interestingly, the PSMA-immunoediting by CART-PSMA cells was more intense in Rag1-KO mice than in WT mice. The lack of endogenous T cells (which compete with the transferred cells for homeostatic cytokines) and T regulatory cells may have favored the antitumor activity of CART cells.

Given the lack self-tolerance of the hPSMA model, we moved to the murine gp75 model. Importantly, the CART-gp75/STING-L combination significantly restrained tumor progression and enhanced overall survival (figure 2E and online supplemental figure S2B), supporting our previous data with the hPSMA model. Interestingly, only tumor cells from mice treated with CART-gp75 cells, either alone or together with 2′3′-cGAMP, exhibited diminished surface expression of gp75 (figure 2F). Similar data were obtained when total gp75 expression was analyzed (online supplemental figure S2C, D). Therefore, these data indicate that gp75 is targeted by CART-gp75 cells, but not by the 2′3′-cGAMP treatment, providing a scenario in which the immune effects of CART cells and STING-L on tumor cells can be differentially studied.

**Figure 3:**
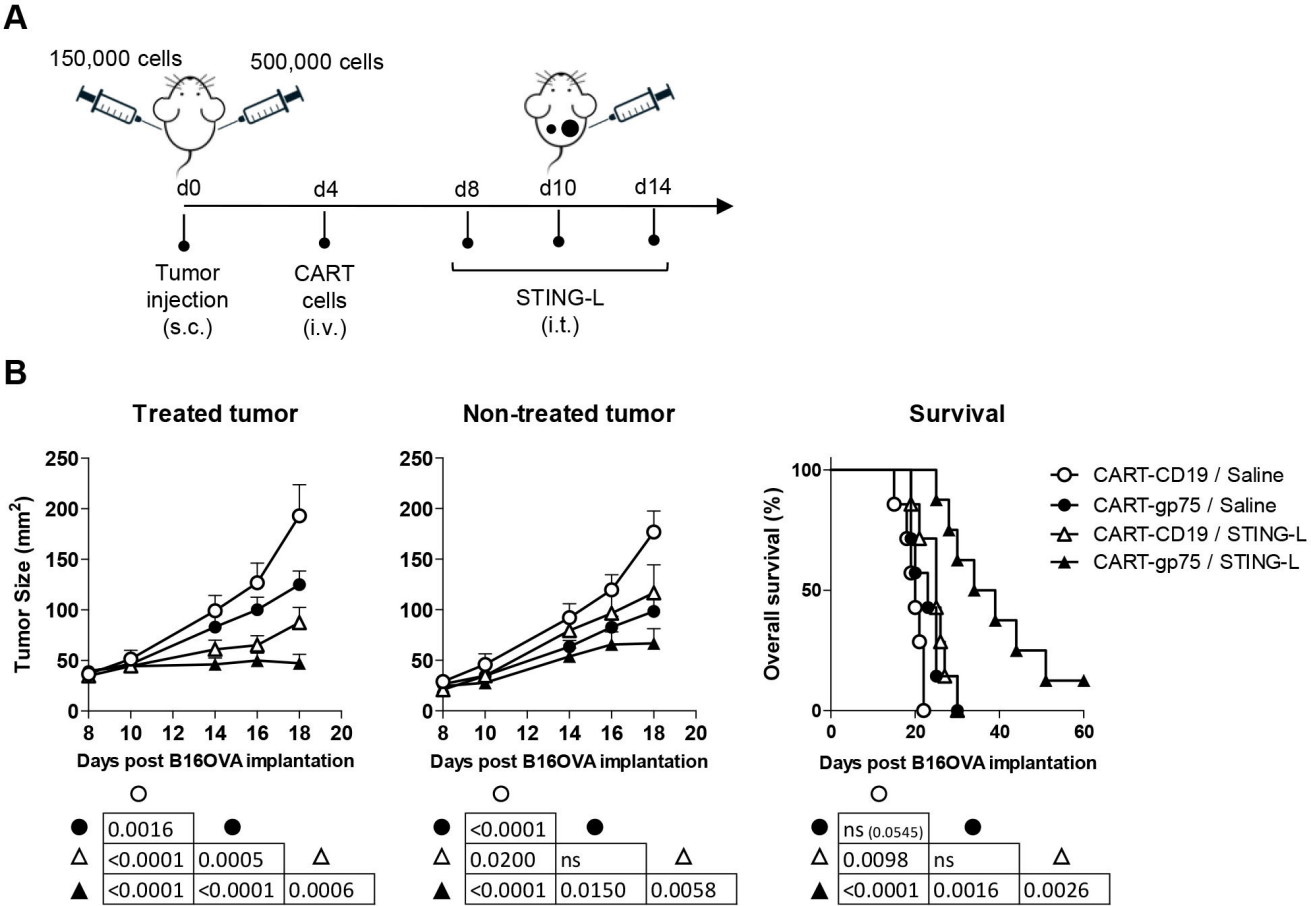
CART therapy along with intratumoral delivery of STING-L controls the growth of distal STING-L-non-treated tumors. (A) Experimental design. To establish B16OVA bilateral tumors, B6 mice were injected subcutaneously with 5×10^5^ and 1.5×10^5^ cells in the right and left flank, respectively. Tumor-bearing mice received an intravenous dose (3×10^6^) of CART-gp75 cells on day 4, along with three intratumoral injections of 2′3′-cGAMP on days 8, 10, and 14 after tumor inoculation (n=8 mice/group). STING-L was injected intratumorally in the larger right tumor. Mice injected with CART-CD19 cells and/or injected intratumorally with saline were used as controls. (B) Tumor growth kinetics of STING-L-treated and STING-L-non-treated tumors (left and middle panels) and survival rate curves (right). The table summarizes the statistical differences across the four groups. Data are represented as mean±SEM (B, left and middle panels). One experiment representative of two experiments. Non-linear regression (curve fit) (B, left and middle panels). Log-rank (Mantel-Cox) test (B, right panel). CART, chimeric antigen receptor T; ns, not significant; PSMA, prostate-specific membrane antigen; STING-L, stimulator of interferon gene ligand.

In order to determine if the CART-cell/STING-L combination was able to control the growth of distal STING-L-non-treated tumors (abscopal effect), mice were inoculated bilaterally with B16OVA cells (3.3 times fewer tumor cells in the case of the left tumor) and treated with CART cells and 2′3′-cGAMP, which was injected only in the right flank tumor (figure 3A). The CART-gp75/STING-L combination significantly restrained progression not only of the treated tumor (right tumor) but also of the contralateral tumor (figure 3B). Furthermore, the antitumor effect also resulted in higher overall survival of the mice receiving the combined treatment. Collectively, all these data suggest a strong synergistic effect between CART cells and 2′3′-cGAMP, with the combination even being able to control distal tumors.

### CART/STING-L combination fosters an endogenous immune response against non-CAR-targeted Ags noticeable also at the systemic level

**Figure 4:**
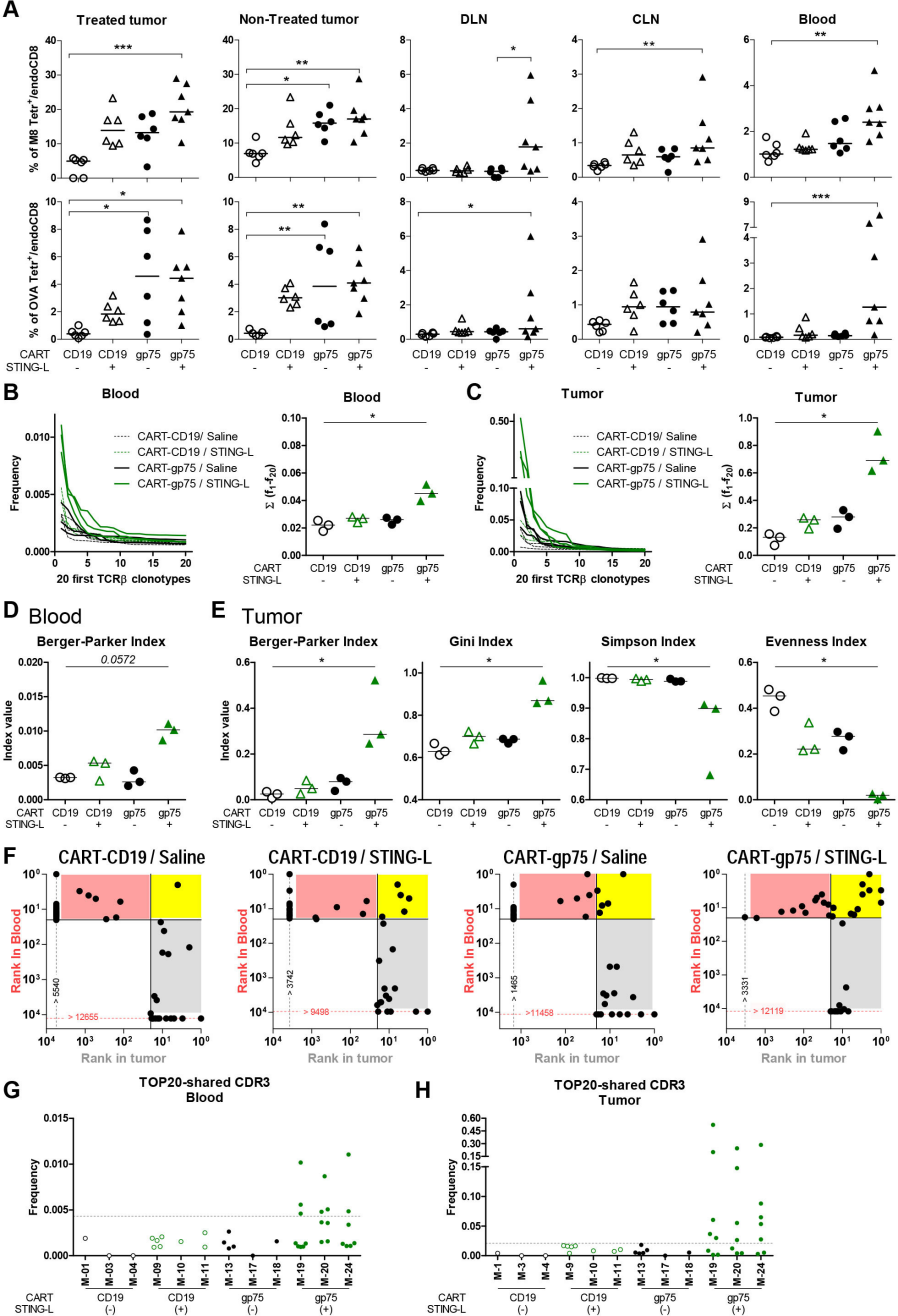
The CART/STING-L combination drives the expansion of a host antitumor T-cell response. (A–C) Mice were treated as in figure 3, except that CART cells were derived from CD45.1 mice. At day 17 of tumor inoculation (days 13 and 9 of ACT and first STING-L injection, respectively), they were bled and sacrificed. (A) Cell suspensions obtained from STING-L-treated and STING-L-non-treated tumors, DLN, CLN and blood were stained and analyzed by flow cytometry. Percentage of M8 (top) and OVA (bottom) tetramer^+^ cells within the endogenous (CD45.2^+^) CD8 T-cell subset. One experiment was representative of at least two experiments. (B–H) Deep bulk CDR3 TCRβ sequencing was performed with gDNA isolated from cell suspensions obtained from peripheral blood and treated tumors (n=3 mice/group). (B, C) Frequency of the 20 most abundant T-cell clones (TOP20 clones) (left) and their cumulative frequency (right) in blood (B) and treated tumor (C). (D, E) Diversity indices were calculated to estimate the clonality of blood (D) and treated tumor TCR repertoire. (F) CDR3β matching between blood and tumor TOP20 clones. The tumor and blood TOP20 clonotypes are plotted according to their rank in tumor (x axis) and blood (y axis) TCR repertoire. Vertical and horizontal dotted lines show the CDR3β detection threshold in tumor and blood, respectively. The CDR3β detection threshold was defined as the highest clonotype rank detected in tumor (gray dotted line) or blood (red dotted line). Those clonotypes that were not detected in tumor or blood were considered to occupy a rank higher than that defined by the threshold and were plotted on the threshold line. The yellow area shows the CDR3β shared by blood and tumor that are among the TOP20 in both tissues (TOP20 best shared CDR3). The gray and pink areas show the shared clonotypes that are among the TOP20 in tumor or in blood, respectively. One mouse representative of each group is shown. (G, H) Frequency of TOP20 best shared CDR3 in blood (G) and tumor (H). Data are represented as median (A, B, right; C, right; D, E). One-way analysis of variance and non-parametric (Kruskal-Wallis) test and Dunns post-test to compare all pairs of groups (A, B, right; C, right; D, E). Only those comparisons with statistical significance are shown. *P<0.05, **P<0.005, ***P<0.0005. CART, chimeric antigen receptor T; CLN, contralateral lymph node; DLN, draining lymph node; STING-L, stimulator of interferon gene ligand.

To evaluate the effect of the combination on the endogenous T-cell response, the percentage of host CD8 T cells specific for two immunodominant CD8 T-cell epitopes of B16OVA tumors, such as the M8 and OVA epitopes, was assessed using H2-K^b^-tetramers in treated and non-treated tumors, LNs (both DLN and CLN (relative to the STING-L-treated tumor)) and peripheral blood. These analyses were performed on day 17 (figure 3A), 3 days after the last STING-L injection. As shown in figure 4A, M8 tetramer^+^ cells predominated over OVA tetramer^+^ cells in tumors. Compared with CART-CD19/saline-treated mice, an increase in the percentage of M8-specific T cells in the treated tumor was observed in all other groups, but this only reached statistical significance in the CART-gp75/STING-L group (figure 4A). The treatment with CART-gp75 cells, either alone or with STING-L, significantly increased the percentage of host CD8 T cells recognizing M8 in the contralateral tumor. Interestingly, mice treated with CART-gp75/STING-L also exhibited a significantly enhanced percentage of M8 tetramer^+^ cells in DLN, CLN and blood. Similar results were observed for host OVA-specific CD8 T cells (figure 4A). These data indicated that the CART/STING-L combination promoted an endogenous CD8 T-cell response against non-CAR-targeted Ags in treated and distal tumors and, importantly, at the systemic level.

We also conducted bulk CDR3 TCRβ sequencing in cells from blood and treated tumors. Regarding the frequency of the 20 most abundant T-cell clonotypes (TOP20 clones), mice treated with the CART-gp75/STING-L combination presented CDR3β clonotypes with a higher frequency than those in the other treatments, both in blood and tumors (figure 4B, C; left), demonstrating a more robust oligoclonal T-cell expansion. Importantly, the cumulative frequency of the TOP20 clones was significantly higher in the CART-gp75/STING-L group as compared with the control group (figure 4B, C; right). A strong correlation was observed between the TOP20 clone cumulative frequency and the percentage of tetramer^+^ cells in total CD3 lymphocytes (online supplemental figure S3A). Among the different diversity indices calculated to estimate the TCR repertoire clonality (online supplemental table S1), only the Berger-Parker Index gave a borderline significant difference in blood (p=0.0572) (online supplemental table S2), with the CART-gp75/STING-L group showing the highest value (increase in dominance) (figure 4D). Regarding the intratumoral TCR repertoire, several diversity indices, including Berger-Parker, Gini, Simpson and Evenness, were significantly altered and their changes indicated greater clonality in the CART-gp75/STING-L group (figure 4E and online supplemental table S3). We also analyzed CDR3β matching between blood and tumor TOP20 clones in each mouse. Certain CDR3β were only found either in tumor or in blood (figure 4F and online supplemental figure S3B). Among the shared CDR3β, some clonotypes showed good ranks (among the TOP20) in both tissues (yellow area, henceforth ‘TOP20-shared CDR3β’), while others were only well ranked either in tumor or in blood (gray and pink areas, respectively). Interestingly, the CART-gp75/STING-L group stood out for presenting TOP20-shared CDR3β clones with a high degree of expansion, both in blood and tumors (figure 4G, H). Together, these data strengthened the idea that the CART/STING-L combination drove the expansion of a host antitumor T-cell response.

### CART-gp75/STING-L combination enhances the infiltration and activation of CART and endogenous T cells in STING-L-treated and STING-L-non-treated tumors

We also analyzed the effects of the treatments on the numbers of CART cells and compared the data with that of total CD45^+^ and endogenous CD8 T (endo-CD8) cells. We noted a generalized increase (as compared with the control group) in the number of CD45^+^ cells infiltrating the tumors and DLN, which reached statistical significance in the treated and non-treated tumors and DLN of the combination group, and non-treated tumors and DLN of the CART-gp75/saline group (online supplemental figure S4A). No significant changes were observed in either CLN or blood, although the number of CD45^+^ cells in blood was slightly lower in the groups receiving CART-gp75 cells.

Regarding CART cells, CD8^+^ cells stood out over CD4^+^ cells in all groups and tissues studied (online supplemental figure S4B). Interestingly, treatment with CART-gp75/STING-L enhanced the number of CD8 and CD4 CART cells infiltrating treated and non-treated tumors, whereas treatment with CART-gp75/saline increased that of CART cells but only in non-treated tumors. No significant changes were observed in tumors from the CART-CD19/STING-L group. These results suggested that while cognate CART cells can be recruited in small tumors (left tumors), local STING-L-derived signals are key for cognate CART cells to be recruited into large tumors (right tumors). A rise in the number of endo-CD8 cells was also observed in treated and non-treated tumors of all treated groups, and this increase was more noticeable in the combination group. These changes were associated with control of tumor growth (online supplemental figure S4C).

In DLN, only a slight increase in the number of CART and endo-CD8 cells was observed in the CART-gp75/saline group, and in that of endo-CD8 cells in the CART-CD19/STING-L group (online supplemental figure S4B). Curiously, CART cells seemed to be excluded from CLN in all treated groups. In addition, a mild decrease in the number of circulating CD8 CART and endo-CD8 cells was observed in all treated mice, with this effect being more noticeable in the combination group. The lower number of CART and endo-CD8 cells in CLN and blood could be due to the improved recruitment of these populations in tumors and DLN.

We also analyzed the expression of several markers associated with effector T-cell trafficking to tumors (CXCR3), activation (CD25 and CD137), exhaustion (PD-1), and effector functions (granzyme B (GzB)) in tumor-infiltrating CART cells, total and tumor-specific endogenous CD8 T cells (endo-CD8 and M8 tetr^+^ cells, respectively) (online supplemental figure 5A, B). The expression levels of CXCR3, CD137, PD-1 and GzB were lower in CART cells than in endo-CD8 and Tetr^+^ cells, and the opposite happened with CD25. Importantly, intratumoral delivery of STING-L increased the expression of CXCR3 in CART cells and endo-CD8 cells. This increase was statistically significant in CD8 CART and endo-CD8 cells and more noticeable in treated tumors.

In addition, CD8 and CD4 CART-gp75 cells had higher levels of CD25 and CD137 than CART-CD19 cells (online supplemental figure S5A, B). In the case of CD137, the combination with STING-L further enhanced the expression in cognate CART cells. In treated tumors, PD1 levels were higher in CD8 CART-gp75 cells than in their CART-CD19 counterparts. In the case of the untreated tumor, only CD8 CART-gp75 cells from the combination group exhibited enhanced expression of PD-1. Moreover, GzB levels were also markedly augmented in cognate CART cells but only in the combination group, with the highest values being detected in the CD8 subset. Expression of CD25, CD137, PD-1, and GzB was hardly detectable in CART cells of DLN, CLN and blood (data not shown). These findings suggested an Ag-driven activation of CART-gp75 cells in treated and non-treated tumors. Interestingly, the activation status of cognate CART cells was fostered by STING-L-derived signals, as shown by the enhanced expression of CD137 and GzB on CART cells from the CART-gp75/STING-L group.

Curiously, endo-CD8 cells from groups treated with CART-gp75 cells also exhibited augmented expression of CD25 (online supplemental figure S5A, B). As for the effects of the treatments on the Tetr^+^ cell phenotype, an enhanced expression of CXCR3, CD25, and GzB was also observed in this cell subset. Again, the increase was more significant in the combination group and in the treated tumors. In summary, the CAR-gp75/STING-L combination enhanced the number of tumor-infiltrating CART and endogenous T cells in both STING-L-treated and STING-L-non-treated tumors. STING-L and CART cells acted synergistically in boosting the activation of CART and endogenous T cells.

### BATF3 is necessary for the CART/STING-L-driven tumor control and epitope spreading

**Figure 5:**
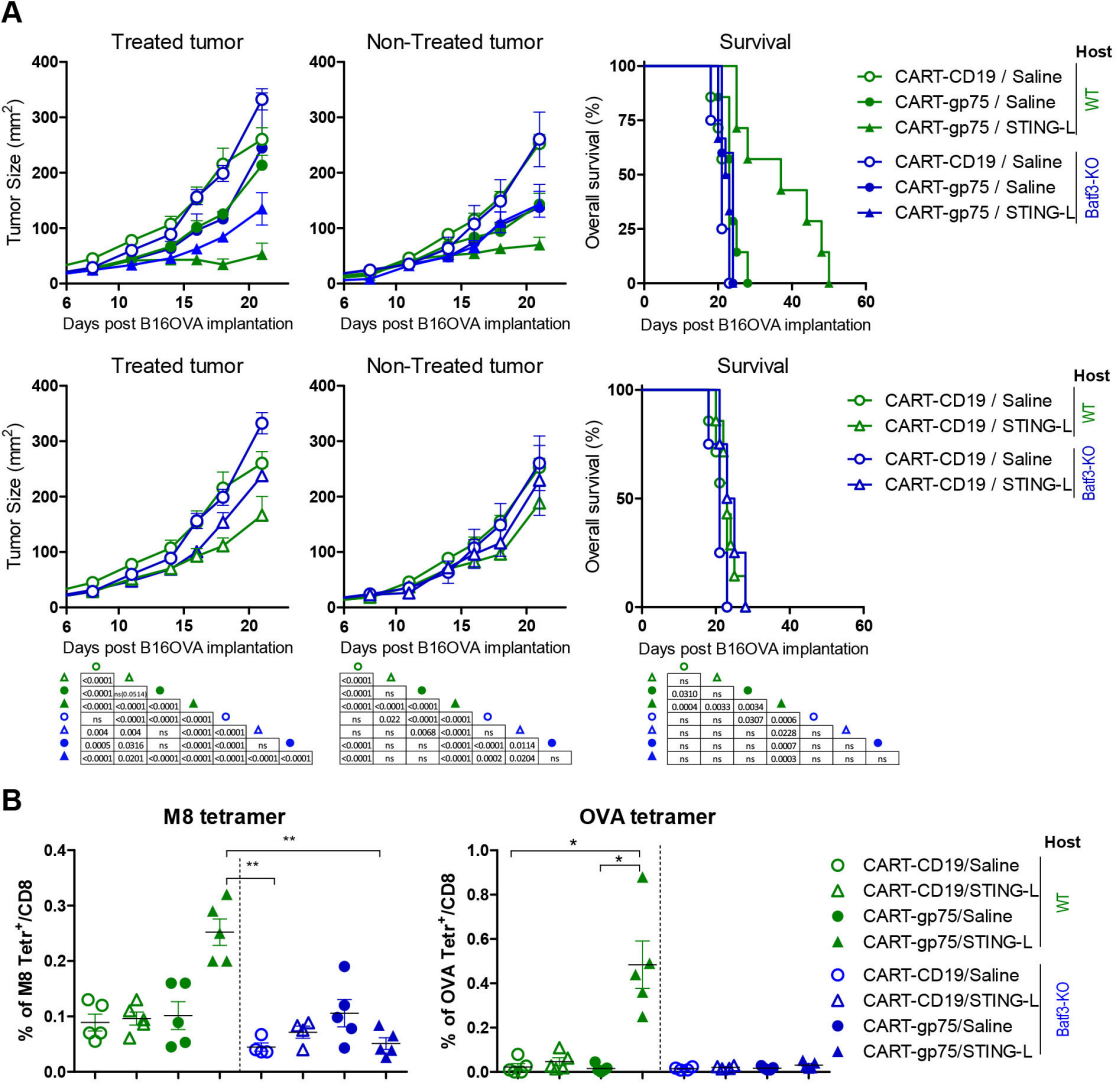
CART/STING-L-mediated local and abscopal therapeutic effect and Ag spreading rely on host Batf3-dependent DCs. WT and Batf3-KO mice were injected subcutaneously with 5×10^5^ and 1.5×10^5^ tumor cells in the right and left flanks, respectively, and treated with CART cells generated from WT mice as in figure 3. (A) Tumor growth kinetics of STING-L-treated and STING-L-non-treated tumors (left and middle panels) and survival rate curves (right panel). For clarity, upper panels show CART-CD19/saline (n=7 WT mice, 5 KO mice), CART-gp75/saline (n=7 WT mice, 5 KO mice) and CART-gp75/STING-L (n=7 WT mice, 6 KO mice) groups, and lower panels show CART-CD19/saline (again) and CART-CD19/STING-L (n=7 WT mice, 5 KO mice) groups. The table summarizes the statistical differences across the eight groups. (B) Percentage of M8 (left) and OVA (right) tetramer^+^ cells within endogenous peripheral blood CD8 T cells on day 14 of tumor implantation. Mice were bled before the third STING-L injection. Data are represented as mean±SEM (A, left and middle panels) or median (B). Non-linear regression (curve fit) (A, and middle panels). Log-rank (Mantel-Cox) test (A, right panels). One-way analysis of variance and non-parametric (Kruskal-Wallis) test and Dunns post-test were used to compare all pairs of groups (B). CART, chimeric antigen receptor T; DC, dendritic cell; KO, knockout; ns, not significant; STING-L, stimulator of interferon gene ligand; WT, wild type.

To ascertain whether Batf3-dependent DCs were involved in the combination-mediated antitumor responses, we used the Batf3-KO strain as recipient mice. Notably, the effect of the combination on tumor growth was impaired in Batf3-KO mice, particularly in non-treated tumors, where only the mild effect of CART-gp75 cells was sizeable (figure 5A). Accordingly, the enhanced overall survival of mice receiving the combination therapy was totally abrogated in Batf3-KO mice. In order to examine the effect of CD103^+^ DC deficiency on the endogenous T-cell response, we measured the frequency of M8 and OVA tetramer^+^ cells in the blood of treated mice. The combination-mediated induction of an endogenous tumor-specific response was completely abolished in Batf3-KO mice (figure 5B). Overall, these results indicate that the local and abscopal antitumor efficacy and the epitope spreading effect of the CART/STING-L combination relies on host CD103^+^ DCs.

### STING signaling, both in the host and in CART cells, is necessary for the CART/STING-L antitumor effect

**Figure 6:**
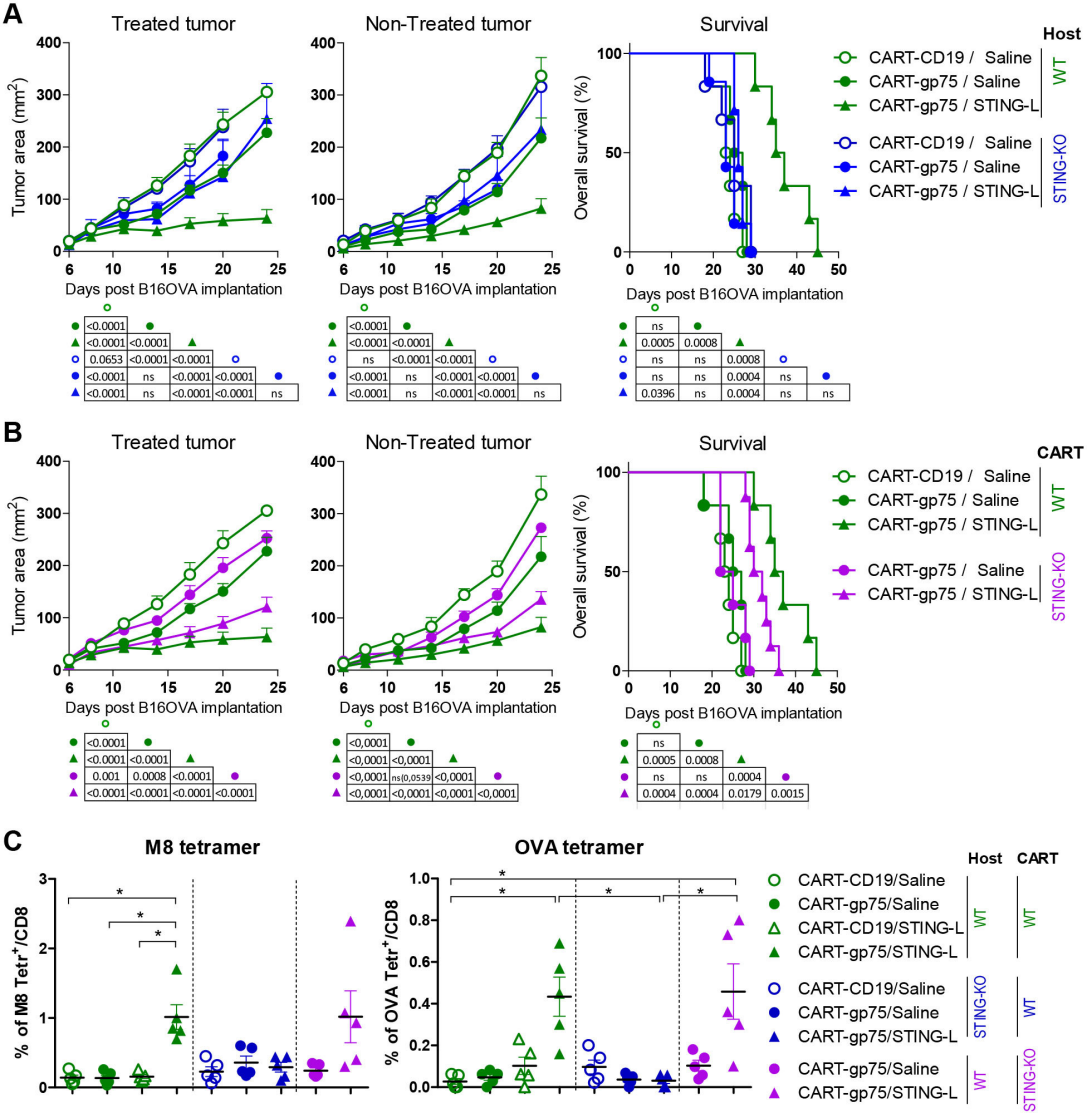
STING signaling, both in the host and in CART cells, is necessary for the local and abscopal antitumor effect of the CART/STING-L combination. (A) Effect of STING signaling in host cells. WT and STING-KO mice bearing bilateral tumors (host) were treated with the WT CART-CD19/saline combination or with WT CART-gp75 cells along with either saline or STING-L as in figure 3 (n=6 WT host mice/group, and n=7 KO host mice/group). (B) Effect of STING signaling on CART cells. In the same experiment as in A; WT mice (n=7 mice/group) bearing bilateral tumors were treated with STING-KO CART-gp75 cells together with either saline or STING-L. (A, B) To simplify the experiment, the groups treated with WT CART-CD19/STING-L combination and with STING-KO CART-CD19 cells along with either saline or STING-L were not included. WT tumor-bearing mice treated with WT CART-CD19/saline and with WT CART-gp75 cells along with either saline or STING-L are repeated (A, B). Tumor growth kinetics of STING-L-treated and STING-L-non-treated tumors (A, B; left and middle panels) and survival rate curves (A, B; right panels). (C) Percentage of M8 (left) and OVA (right) tetramer^+^ cells within endogenous peripheral blood CD8 T cells on day 14 of tumor implantation as in figure 5. Data are represented as mean±SEM (A, B; left and middle panels) or median (C). Non-linear regression (curve fit) (A, B; left and middle panels). Log-rank (Mantel-Cox) test (A and B, right panels). One-way analysis of variance and non-parametric (Kruskal-Wallis) test and Dunns post-test to compare all pairs of groups (C). *P<0.05. One experiment was representative of two experiments. CART, chimeric antigen receptor T; KO, knockout; ns, not significant; STING-L, stimulator of interferon gene ligand; WT, wild type.

To determine whether the host STING pathway was required for the antitumor activity of the CART/STING-L treatment, we compared the therapeutic efficacy of the combination in wild type (WT) and STING-KO tumor-bearing mice. As depicted in figure 6A, tumor growth delay due to CART/STING-L therapy was completely lost in treated and non-treated tumors of STING-KO mice, in which only the modest effect of CART-gp75 cells was noted. Only WT mice treated with the combination showed prolonged survival.

To ascertain whether the efficacy of the treatment was also dependent on CART intrinsic STING signaling, we generated STING-deficient CART-gp75 cells. As depicted in figure 6B, the mild effect of CART-gp75 cells on tumor growth was impaired when they were STING deficient. The STING-KO CART-driven combination substantially controlled the growth of treated and non-treated tumors, although less efficiently than when the combination was driven by WT cells. Consistently, only the combined treatment groups exhibited enhanced survival, although to a lesser extent when STING-KO CART-gp75 cells were used.

We also measured the STING signaling effect on the induction of an endogenous tumor-specific T-cell response. The enhanced percentage of circulating host tumor-specific T cells observed after the WT CART-driven combination was completely abolished when STING-KO mice were used as recipients (figure 6C). However, STING deficiency in CART cells did not impair the combination-mediated priming of endogenous tumor-specific T cells. Collectively, our data suggest that STING signaling, both in the host and in CART cells, is necessary for the local and abscopal effect of the CART/STING-L combination. However, different mechanisms seem to be behind these outcomes, such as the abolition of the Ag spreading effect when STING signaling is absent in the host, or a decreased antitumor efficacy of CART cells, when STING signaling is deficient in transferred T cells.

### Perf deficiency in CART cells impairs the epitope spreading effect and the antitumor efficacy of the CART/STING-L combination

**Figure 7:**
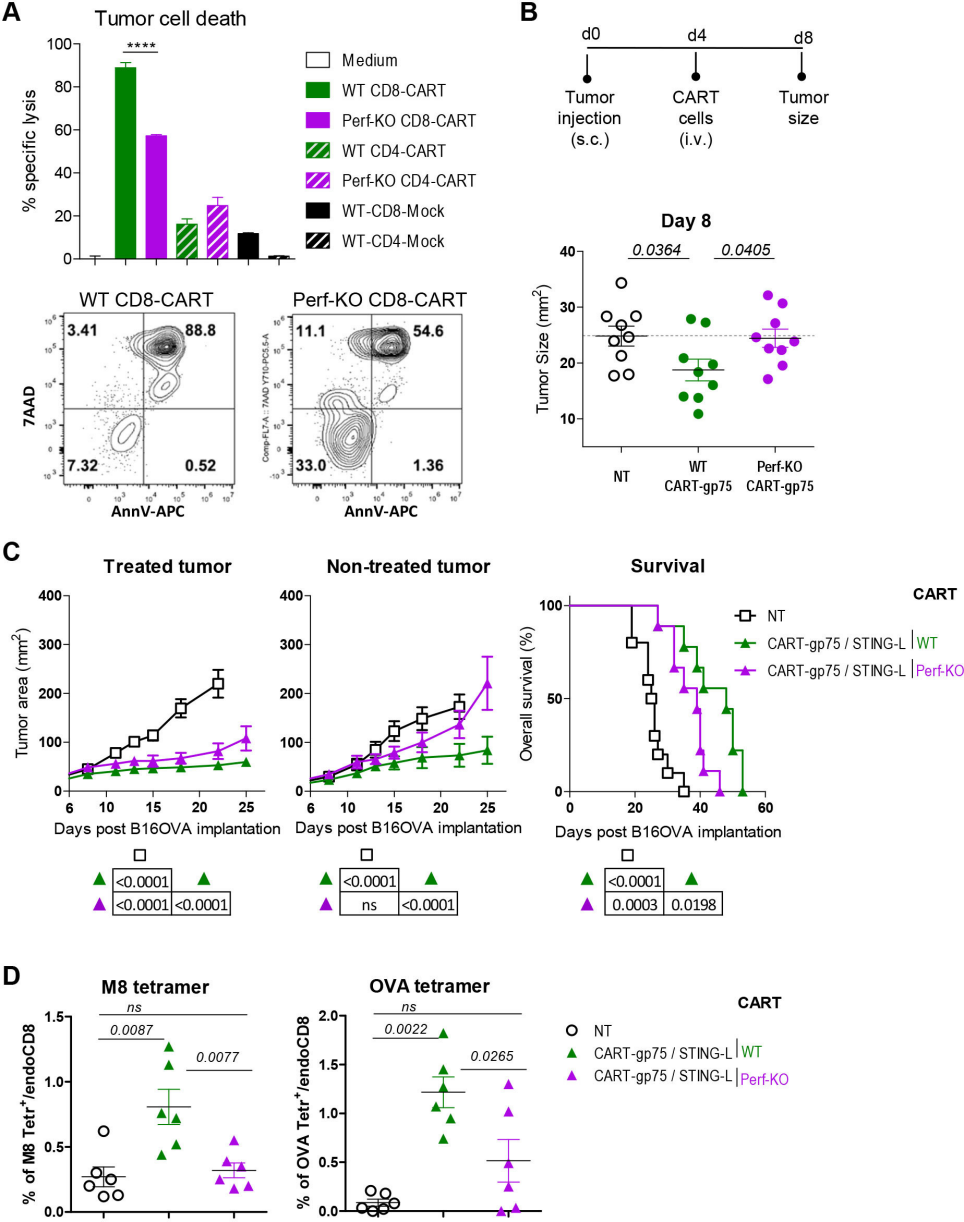
Perf-deficiency in CART cells impaired the Ag spreading and the local and abscopal effects of CART/STING-L combination. (A) WT or Perf-KO CD8 or CD4 T cells expressing CART-PSMA or CART-CD19 (mock) were cultured in vitro with B16-PSMA tumor cells at a ratio of 1:1 (CART cell:tumor cell) (test wells). As a control, tumor cells were cultured alone (medium, ctrl wells). The percentage of dead tumor cells (7AAD^+^CD45^−^) in total tumor cells (CD45^−^) was analyzed 12 hours later by flow cytometry. The graph shows the killing activity of T cells as percentage of specific lysis calculated as described in the Methods section. Representative flow cytometry dot plots showing percentage of dead tumor cells in the culture conditions with WT or Perf-KO CART-PSMA CD8 T cells are also included. (B, C) WT mice (n=10 mice/group) bearing bilateral tumors were left NT or received an intravenous dose of WT or Perf-KO CART-gp75 cells along with STING-L as in figure 3. (B) Size of right tumor on day 4 of ACT (day 8 of tumor implantation) before STING-L injection. (C) Tumor growth kinetics of STING-L-treated and STING-L-non-treated tumors (left and middle panels) and survival rate curves (right). (D) Percentage of M8 (left) and OVA (right) tetramer^+^ cells within endogenous peripheral blood CD8 T cells on day 14 of tumor implantation as in figure 5. (A) One experiment was representative of two experiments. (B–D) Two compiled experiments. Data are represented as mean±SD (A) and mean±SEM (B, C, left and middle panels; D). Non-parametric Mann-Whitney test, two-tailed (A, B, D). Non-linear regression (curve fit) (C, left and middle panels). Log-rank (Mantel-Cox) test (C, right panel). ACT, adoptive T-cell transfer; Ag, antigen; CART, chimeric antigen receptor T; KO, knockout; ns, not significant; NT, untreated; PSMA, Perf, perforin; PSMA, prostate-specific membrane antigen; STING-L, stimulator of interferon gene ligand; WT, wild type.

CD8 T-cell cytotoxic activity induces tumor-cell death and promotes the priming of tumor-specific T cells,^31 32^ with this effect being partially dependent on CD8 T-cell Perf-mediated killing of tumor cells.^32^ In order to determine if Perf in CART cells was required for the antitumor efficacy of the CART/STING-L combination, we generated Perf-deficient CART cells and compared them with WT CART cells. In vitro, Perf-KO CD8 CART cells were less efficient than their WT counterparts in killing tumor cells (figure 7A). As was found in previous studies,^33 34^ CD4 CART cells were much less efficient at killing target cells than their CD8 counterparts and were not dependent on Perf. When injected into 4-day B16OVA-tumor bearing mice, Perf-KO CART-gp75 cells did not delay early tumor growth (figure 7B). The combination treatment driven by Perf-KO CART cells substantially controlled the growth of the treated tumors, although less efficiently than when the combination was driven by WT cells (figure 7C). Interestingly, the loss of Perf-mediated cytotoxicity by CART cells more seriously affected the abscopal effect of the CART/STING-L combination. The combined treatment improved survival, although to a lesser extent when CART-gp75 cells were Perf-deficient. Notably, the percentage of circulating host tetramer^+^ T cells was significantly lower in mice that received the combination with Perf-KO CART cells (figure 7D). In summary, the failure of Perf-mediated cytotoxicity by CART cells impaired the Ag spreading and the local and abscopal effects of the CART/STING-L combination.

### Both CART cells and the STING-L-induced immune response are necessary for long-term control of distal tumor growth and reducing physical deterioration (PD)-associated diseases

We also analyzed the death cause of tumor-bearing mice treated with CART/STING-L therapy through all the experiments done. PD is common in subcutaneous B16 tumor models, and mice with this symptom must be euthanized before reaching oncological experiment endpoints. This is mainly due to cachexia, which in turn is mediated by tumor growth-driven inflammation and muscle wasting.^35^ Interestingly, when WT tumor-bearing mice were treated with WT CART cells, the CART-gp75/STING-L combination not only did slow down tumor growth (figure 3B) but also markedly reduced the euthanasia cases due to PD (online supplemental figure S6), with both effects contributing to increase survival. In WT mice treated with CART-CD19/STING-L or CART-gp75/saline, the proportion of PD-associated deaths decreased but to a lesser extent, indicating that CART-gp75 cells and STING-L acted synergistically, controlling PD-associated disease. Regarding tumor growth-associated deaths, the deceased by the left tumor outgrowth predominated over those by right tumor outgrowth in mice treated with CART-CD19/STING-L, while the opposite occurred in the CART-gp75/saline group. The deceased proportion due to right and left tumor outgrowth was equal in mice treated with the combination. These results indicated that while STING-L treatment favored the long-term control of right tumor growth, CART-gp75 cells did so in left tumors, probably because they were smaller.

When the CART/STING-L approach was carried out in Batf3-KO mice, the effects of STING-L were lost. Interestingly, the proportion of deaths due to left tumor outgrowth increased in BATF3-KO mice treated with CART-gp75/Saline, as compared with WT mice treated similarly. Importantly, a similar effect was observed in STING-KO tumor-bearing mice treated with WT CART-gp75 plus saline. These results suggested that a part of the enhanced control of distal tumor by the CART-gp75/saline therapy was due to the STING-L-induced, Batf3 DC-mediated host immune response.

When CART cells were STING-L-deficient, the death proportion due to distal tumor outgrowth increased, reaffirming the impaired antitumor efficacy of STING-KO CART cells and the key role of CART-gp75 cells in the long-term control of small tumors. Finally, we noted an increase in the number of PD-associated deaths when the CART/STING-L treatment was performed with STING-KO and Perf-KO CART-gp75 cells, as compared with those observed when the combination was run with WT CART-gp75. However, this increase did not reach the levels of PD-associated deaths in control mice (WT tumor-bearing mice treated with WT CART-CD19 cells plus saline). This highlighted the synergistic action of CART-gp75 cells and STING-L-mediated response on controlling PD-associated disease.

## Discussion

Loss or diminished expression of the target Ag is an adaptive resistance mechanism affecting the efficacy of CART-cell therapy. Our preclinical models recapitulate this phenomenon as shown by the diminished target Ag expression levels in tumors treated with cognate CART cells. The aim of this study was to counteract target Ag escape by combining CART-cell therapy with an immunomodulatory agent able to evoke an endogenous antitumor immune response. We have shown that the combination of CART cells with the intratumoral delivery of 2′3′-cGAMP fostered a T-cell response against non-CAR-targeted Ags. This manifested as an increased percentage of tumor-specific endogenous T cells, identified by tetramer staining. The oligoclonal expansion of host T cells, as revealed by in-depth analysis of the TCRβ repertoire, confirmed this finding.

The combination therapy restrained tumor progression of STING-L-treated and STING-L-non-treated tumors. Of the two CAR-target Ag models used, the gp75 model is characterized by having a low surface Ag density. Therefore, the results observed in this model highlight the translational relevance of this strategy. Importantly, the epitope spreading correlated with the efficacy of the combined treatment and both phenomena fully depended on host STING signaling and Batf3-dependent DCs (online supplemental figure S7). The effectiveness of the treatment also partially depended on the release of Perf by CART cells. Interestingly, Perf deficiency in CART cells also significantly diminished the epitope spreading effect of the CART/STING-L combination. These data reveal the dual role of the cytotoxic activity of CART cells in the context of CART/STING-L therapy: on one hand, its implication in the direct destruction of tumor cells and, on the other hand, in the release of Ags necessary for the epitope spreading effect. The fact that Perf deficiency did not eliminate completely the cytotoxic activity of CART cells (as shown in the in vitro assays) and the epitope spreading suggests that other T cell-mediated tumor-cell death pathways may be involved.^36^ Furthermore, we cannot rule out a direct effect of STING-L on the killing of tumor cells,^37^ which may also have favored the release of tumor Ags.

Similarly to treatment with STING-L alone, cognate CART cells in monotherapy also increased the percentage of tumor-specific endogenous T cells in the treated and contralateral tumor, although less noticeably than the combined treatment. Our data confirmed previous evidence that CART-cell therapy also induces epitope spreading.^10 11^ It has been reported that CD8 T-cell cytotoxic activity induced immunogeneic tumor-cell death (ITCD),^31 32^ with this phenomenon being partially dependent on Perf-dependent killing.^32^ This CART-mediated ITCD may explain the enhanced tumor-specific endogenous T-cell response observed with CART cell monotherapies. However, the Ag spreading derived directly from the action of STING-L or cognate CART cells alone did not achieve a meaningful level for therapeutic efficacy. Importantly, only in the combination group did the activation of endogenous T cells translate into a systemic response detectable in blood. The presence of circulating tumor-specific T cells explain the efficient control of distal tumors in the CART/STING-L group. Our findings suggest that the joint action of CART cells, destroying tumor cells and releasing secondary Ags, together with the immune-stimulatory action of STING-L, is critical to induce a tumor-specific endogenous T-cell response strong enough to control local and distal tumors. Other studies have also shown the importance of combining adjuvants, such as IL-12, 41BB agonist or pathogen-based vaccines, with an ACT regimen to enhance the epitope spreading^14 15^ and the local and abscopal^15^ effects mediated by transferred cells.

The enhanced tumor control of the combined treatment was also associated with an increase in the number of CART cells and endogenous T cells into the treated and distal tumors. Interestingly, STING-L treatment also upregulated the expression of the IFN-inducible chemokine receptor CXCR3 in CART and endo-CD8 cells from treated and distal tumors. Our results confirm recent findings by Xu *et al*, who demonstrated the efficacy of the CART/STING-L combination in a unilateral model of locally advanced breast cancer.^38^ These authors have shown that the subcutaneous injection of STING-L at a site remote from the tumor leads to an enhanced expression of CXCL9/10 within the TME and CXCR3 on tumor-infiltrating T cells.^39^ CXCL9/10 chemokines recruit CXCR3-expressing effector T cells^39^ and the CXCL-9–10/CXCR3 axis prompted by STING-L-derived signals may explain the increased numbers of CART and endogenous T cells within the tumors. The enhanced expression of CXCR3 and CXCL9/10 at a distant site from that of injection may be due to a small leakage of STING-L or to the STING-mediated induction of a systemic IFN-I-response.

However, the increase in the number of CART cells was always greater when STING-L was combined with cognate CART cells than with unrelated CART cells. This suggests that other biological processes, such as the activation and proliferation of CART cells on Ag recognition, may have contributed to augment the number of these cells in the tumors. In this context, cognate CART cells exhibited clear signs of activation, as was evident by upregulation of CD25, CD137, PD-1, and GzB. Interestingly, the number and CD25 expression levels of total endogenous T cells infiltrating the tumors were also higher when STING-L was combined with cognate CART cells than with CART-CD19 cells, indicating that the antitumor effect of CART-gp75 cells also favors the activation of endogenous T lymphocytes. The boosting effect of STING-L and cognate CART cells was more pronounced on endogenous tumor-specific T cells, as indicated by the increased number of tumor-infiltrating Tetr^+^ cells and their enhanced expression of CXCR3, CD25, and GzB in the combination group. More importantly, these effects on endogenous T cells were observed in both the STING-L-treated and STING-L-non-treated tumor. Therefore, the induction of a systemic response and the enhanced recruitment and activation of endogenous T cells in distal tumors may account for the abscopal effect of the CART/STING-L combination.

Interestingly, our results indicate that the local and abscopal antitumor effects of the CART/STING-L combination also depend on STING signaling in CART cells. The importance of the direct effects of pharmacological STING activation on the antitumor properties of T cells has been demonstrated by Imanishi *et al*.^28^ Moreover, we have observed that STING-KO CART cells in monotherapy also exhibited an impaired antitumor response. This confirmed recent findings by Li *et al* demonstrating that in the absence of pharmacological activation, STING-proficient CD8 T cells were more effective in ACT schedules than their STING-deficient counterparts.^40^ Further studies are necessary to decipher the role of intrinsic STING signaling on the antitumor activity of transferred T cells.

2′3′-cGAMP properties may not be extrapolated to other pharmacological STING-Ls. Indeed, although 2′3′-cGAMP (poorly membrane permeable) inhibited T-cell proliferation, it did not cause apoptosis.^28^ In contrast, STING activation with cell-permeable small molecules, such as dimethylxanthone acetic acid (DMXAA) and 10-carboxymethyl-9-acridanone, promoted T-cell apoptosis.^29 30 41^ Similar results were observed in T cells with constitutively active STING mutations.^30^ Interestingly, patients carrying constitutive active STING mutations showed reduced numbers of memory T cells.^30^ In summary, depending on the degree and duration of STING activation in T cells, the outcome can be very different. Therefore, the choice of the appropriate STING-L is very important so that the immune-stimulatory functions of STING on the innate immune system can be exploited while preserving T-cell functions.

As already mentioned, we cannot exclude that a small leakage and systemic distribution of STING-L on intratumoral injection had effects on the distal tumor. However, when administered intravenously, 2′3′-cGAMP was reported to exert only a poor antitumor effect.^42^ Furthermore, several clinical trials investigating intravenous administration of DMXAA have also shown disappointing anticancer activity.^43^ Even so, the effects of the systemic application of STING-Ls in combination with CART cell therapy deserve to be investigated. One of the problems with intravenous administration of STING-Ls is their short half-life (especially in the case of 2′3′-cGAMP) due to their sensitivity to degradation by ENPP1.^44^ Packaging of 2′3′-cGAMP into liposomal nanoparticles improves its half-life, tumor penetration, and cellular uptake on intravenous injection, as compared with soluble cGAMP.^42^ On the other hand, new STING-Ls with enhanced cell permeability and resistance to hydrolysis by ENPP1 are being developed.^45^ It would be interesting to test these new STING-L formulations intravenously in combination with CART cells.

Our data indicate that the STING agonist 2′3′-cGAMP is a suitable adjuvant to combine with CART-cell therapy and promote epitope spreading. Recently, another study has used Flt3L-secreting CART cells to expand and differentiate CD103^+^ DC and, thus, extend the immune response to secondary Ags.^46^ However, in this study, the combination of CART cells with other adjuvants, such as poly(I:C) plus anti-4-1BB mAb, was required to activate CD103^+^ DCs and elicit the full benefit of the Flt3L-secreting T cells.

Overall, our observations could help in the development of new therapeutic strategies to combine with CART cells, based on the intratumoral delivery of immunomodulatory agents capable of fostering epitope spreading. An important limitation of our study is the use of tumor cell lines, such as B16-PSMA and B16OVA, whose inherent antigenicity favors the induction of an endogenous immune response. The existence of strong tumor Ags may also help spread the immune response to other weak Ags. In this sense, the strong antigenicity of hPSMA in mice would explain why only in the B16-PSMA model the CART/STING-L treatment induced vitiligo (indicative of epitope spreading to melanocyte Ags) and yielded survivors resistant to B16-F10 rechallenge. Therefore, the efficacy of the CART/STING-L combination in less antigenic tumors remains to be demonstrated. In this context, the combination with approaches favoring the expression of new, and therefore potent, Ag in tumor cells, such as the inhibition of the nonsense-mediated messenger RNA breakdown machinery, may be useful.^47^ Finally, although the first challenges for CART cells in solid tumors are to improve their tumor recruitment, survival and action in the hostile TME, the Ag-loss variant escape resulting from the on-target activity of CART cells will likely be the next barrier to overcome. Therefore, for the success of CART-cell therapy in solid tumors, approaches that overwhelm these first obstacles and that allow boosting a secondary antitumor immune response will be necessary.

## Data Availability

Data are available upon reasonable request. All data relevant to the study are included in the article or uploaded as supplementary information. The data and additional information will be available from Sandra Hervas-Stubbs (mshervas@unav.es) to third parties who reasonably request it.

## References

[1] Majzner RG, Mackall CL. Tumor antigen escape from CAR T-cell therapy. Cancer Discov 2018;8:1219–26. 10.1158/2159-8290.CD-18-0442 30135176

[2] Berger TR, Maus MV. Mechanisms of response and resistance to CAR T cell therapies. Curr Opin Immunol 2021;69:56–64. 10.1016/j.coi.2021.02.010 33752101

[3] Larson RC, Maus MV. Recent advances and discoveries in the mechanisms and functions of CAR T cells. Nat Rev Cancer 2021;21:145–61. 10.1038/s41568-020-00323-z 33483715 PMC8353572

[4] O’Rourke DM, Nasrallah MP, Desai A. A single dose of peripherally infused EGFRvIII-directed CAR T cells mediates antigen loss and induces adaptive resistance in patients with recurrent glioblastoma. Sci Transl Med 2017;9. 10.1126/scitranslmed.aaa0984 PMC5762203 28724573

[5] Schreiber RD, Old LJ, Smyth MJ. Cancer immunoediting: Integrating immunity’s roles in cancer suppression and promotion. Science 2011;331:1565–70. 10.1126/science.1203486 21436444

[6] Hamieh M, Dobrin A, Cabriolu A, et al. CAR T cell trogocytosis and cooperative killing regulate tumour antigen escape. Nature 2019;568:112–6. 10.1038/s41586-019-1054-1 30918399 PMC6707377

[7] Watanabe K, Terakura S, Martens AC, et al. Target antigen density governs the efficacy of anti-CD20-CD28-CD3 ζ chimeric antigen receptor-modified effector CD8+ T cells. J Immunol 2015;194:911–20. 10.4049/jimmunol.1402346 25520398

[8] Vanderlugt CL, Miller SD. Epitope spreading in immune-mediated diseases: implications for immunotherapy. Nat Rev Immunol 2002;2:85–95. 10.1038/nri724 11910899

[9] Sampson JH, Choi BD, Sanchez-Perez L, et al. EGFRvIII mCAR-modified T-cell therapy cures mice with established intracerebral glioma and generates host immunity against tumor-antigen loss. Clin Cancer Res 2014;20:972–84. 10.1158/1078-0432.CCR-13-0709 24352643 PMC3943170

[10] Heckler M, Dougan SK. Unmasking pancreatic cancer: epitope spreading after single antigen chimeric antigen receptor T-cell therapy in a human phase I trial. Gastroenterology 2018;155:11–14. 10.1053/j.gastro.2018.06.023 29885301

[11] Beatty GL, Haas AR, Maus MV, et al. Mesothelin-specific chimeric antigen receptor mRNA-engineered T cells induce anti-tumor activity in solid malignancies. Cancer Immunol Res 2014;2:112–20. 10.1158/2326-6066.CIR-13-0170 24579088 PMC3932715

[12] Hont AB, Cruz CR, Ulrey R, et al. Immunotherapy of relapsed and refractory solid tumors with ex vivo expanded multi-tumor associated antigen specific cytotoxic T lymphocytes: a phase I study. J Clin Oncol 2019;37:2349–59. 10.1200/JCO.19.00177 31356143 PMC6804838

[13] Leung W, Heslop HE. Adoptive immunotherapy with antigen-specific T cells expressing a native TCR. Cancer Immunol Res 2019;7:528–33. 10.1158/2326-6066.CIR-18-0888 30936089 PMC6462216

[14] Xin G, Khatun A, Topchyan P, et al. Pathogen-boosted adoptive cell transfer therapy induces endogenous antitumor immunity through antigen spreading. Cancer Immunol Res 2020;8:7–18. 10.1158/2326-6066.CIR-19-0251 31719059 PMC6946848

[15] Etxeberria I, Bolaños E, Quetglas JI, et al. Intratumor adoptive transfer of IL-12 mRNA transiently engineered antitumor CD8^+^ T cells. Cancer Cell 2019;36:613–29. 10.1016/j.ccell.2019.10.006 31761658

[16] Chapuis AG, Roberts IM, Thompson JA, et al. T-cell therapy using interleukin-21-primed cytotoxic T-cell lymphocytes combined with cytotoxic T-cell lymphocyte antigen-4 blockade results in long-term cell persistence and durable tumor regression. J Clin Oncol 2016;34:3787–95. 10.1200/JCO.2015.65.5142 27269940 PMC5477923

[17] Broz ML, Binnewies M, Boldajipour B, et al. Dissecting the tumor myeloid compartment reveals rare activating antigen-presenting cells critical for T cell immunity. Cancer Cell 2014;26:638–52. 10.1016/j.ccell.2014.09.007 25446897 PMC4254577

[18] Edelson BT, KC W, Juang R, et al. Peripheral CD103+ dendritic cells form a unified subset developmentally related to CD8α+ conventional dendritic cells. J Exp Med 2010;207:823–36. 10.1084/jem.20091627 20351058 PMC2856032

[19] Engelhardt JJ, Boldajipour B, Beemiller P, et al. Marginating dendritic cells of the tumor microenvironment cross-present tumor antigens and stably engage tumor-specific T cells. Cancer Cell 2012;21:402–17. 10.1016/j.ccr.2012.01.008 22439936 PMC3311997

[20] Fuertes MB, Kacha AK, Kline J, et al. Host type I IFN signals are required for antitumor CD8+ T cell responses through CD8α+ dendritic cells. J Exp Med 2011;208:2005–16. 10.1084/jem.20101159 21930765 PMC3182064

[21] Roberts EW, Broz ML, Binnewies M, et al. Critical role for CD103(+)/CD141(+) dendritic cells bearing CCR7 for tumor antigen trafficking and priming of T cell immunity in melanoma. Cancer Cell 2016;30:324–36. 10.1016/j.ccell.2016.06.003 27424807 PMC5374862

[22] Woo S-R, Fuertes MB, Corrales L, et al. STING-dependent cytosolic DNA sensing mediates innate immune recognition of immunogenic tumors. Immunity 2014;41:830–42. 10.1016/j.immuni.2014.10.017 25517615 PMC4384884

[23] Corrales L, Glickman LH, McWhirter SM, et al. Direct activation of sting in the tumor microenvironment leads to potent and systemic tumor regression and immunity. Cell Rep 2015;11:1018–30. 10.1016/j.celrep.2015.04.031 25959818 PMC4440852

[24] Pastor F, Kolonias D, McNamara JO, et al. Targeting 4-1BB costimulation to disseminated tumor lesions with Bi-specific oligonucleotide aptamers. Mol Ther 2011;19:1878–86. 10.1038/mt.2011.145 21829171 PMC3188744

[25] Williams MR, DeSpenza T, Li M, et al. Hyperactivity of newborn PTEN knock-out neurons results from increased excitatory synaptic drive. J Neurosci 2015;35:943–59. 10.1523/JNEUROSCI.3144-14.2015 25609613 PMC4300333

[26] Naftzger C, Takechi Y, Kohda H, et al. Immune response to a differentiation antigen induced by altered antigen: a study of tumor rejection and autoimmunity. Proc Natl Acad Sci U S A 1996;93:14809–14. 10.1073/pnas.93.25.14809 8962137 PMC26218

[27] Mintz B, Silvers WK. ‘Intrinsic’ immunological tolerance in allophenic mice. Science 1967;158:1484–7. 10.1126/science.158.3807.1484 6058691

[28] Imanishi T, Unno M, Kobayashi W, et al. Reciprocal regulation of sting and TCR signaling by mTORC1 for T-cell activation and function. Life Sci Alliance 2019;2:e201800282. 10.26508/lsa.201800282 30683688 PMC6348487

[29] Larkin B, Ilyukha V, Sorokin M, et al. Cutting edge: activation of sting in T cells induces type I IFN responses and cell death. J Immunol 2017;199:397–402. 10.4049/jimmunol.1601999 28615418 PMC5525333

[30] Wu J, Chen Y-J, Dobbs N, et al. STING-mediated disruption of calcium homeostasis chronically activates ER stress and primes T cell death. J Exp Med 2019;216:867–83. 10.1084/jem.20182192 30886058 PMC6446864

[31] Minute L, Teijeira A, Sanchez-Paulete AR, et al. Cellular cytotoxicity is a form of immunogenic cell death. J Immunother Cancer 2020;8:e000325. 10.1136/jitc-2019-000325 32217765 PMC7206966

[32] Jaime-Sanchez P, Uranga-Murillo I, Aguilo N, et al. Cell death induced by cytotoxic CD8^+^ T cells is immunogenic and primes caspase-3-dependent spread immunity against endogenous tumor antigens. J Immunother Cancer 2020;8:e000528. 10.1136/jitc-2020-000528 32241808 PMC7174069

[33] Stalder T, Hahn S, Erb P. Fas antigen is the major target molecule for CD4+ T cell-mediated cytotoxicity. J Immunol 1994;152:1127–33. 7507960

[34] Liadi I, Singh H, Romain G, et al. Individual motile CD4(+) T cells can participate in efficient multikilling through conjugation to multiple tumor cells. Cancer Immunol Res 2015;3:473–82. 10.1158/2326-6066.CIR-14-0195 25711538 PMC4421910

[35] Voltarelli FA, Frajacomo FT, Padilha CdeS, et al. Syngeneic B16F10 melanoma causes cachexia and impaired skeletal muscle strength and locomotor activity in mice. Front Physiol 2017;8:715. 10.3389/fphys.2017.00715 29033844 PMC5626871

[36] Martínez-Lostao L, Anel A, Pardo J. How do cytotoxic lymphocytes kill cancer cells? Clin Cancer Res 2015;21:5047–56. 10.1158/1078-0432.CCR-15-0685 26567364

[37] Tang C-HA, Zundell JA, Ranatunga S, et al. Agonist-mediated activation of STING induces apoptosis in malignant B cells. Cancer Res 2016;76:2137–52. 10.1158/0008-5472.CAN-15-1885 26951929 PMC4873432

[38] Xu N, Palmer DC, Robeson AC, et al. STING agonist promotes CAR T cell trafficking and persistence in breast cancer. J Exp Med 2021;218. 10.1084/jem.20200844 PMC7780733 33382402

[39] Spranger S, Dai D, Horton B, et al. Tumor-residing Batf3 dendritic cells are required for effector T cell trafficking and adoptive T cell therapy. Cancer Cell 2017;31:711–23. 10.1016/j.ccell.2017.04.003 28486109 PMC5650691

[40] Li W, Lu L, Lu J, et al. cGAS-STING-mediated DNA sensing maintains CD8^+^ T cell stemness and promotes antitumor T cell therapy. Sci Transl Med 2020;12 10.1126/scitranslmed.aay9013 32581136

[41] Gulen MF, Koch U, Haag SM, et al. Signalling strength determines proapoptotic functions of STING. Nat Commun 2017;8:427. 10.1038/s41467-017-00573-w 28874664 PMC5585373

[42] Cheng N, Watkins-Schulz R, Junkins RD, et al. A nanoparticle-incorporated STING activator enhances antitumor immunity in PD-L1-insensitive models of triple-negative breast cancer. JCI Insight 2018;3:1. 10.1172/jci.insight.120638 PMC6302949 30429378

[43] Lara PN, Douillard J-Y, Nakagawa K, et al. Randomized phase III placebo-controlled trial of carboplatin and paclitaxel with or without the vascular disrupting agent vadimezan (ASA404) in advanced non-small-cell lung cancer. J Clin Oncol 2011;29:2965–71. 10.1200/JCO.2011.35.0660 21709202

[44] Onyedibe KI, Wang M, Sintim HO. ENPP1, an old enzyme with new functions, and small molecule inhibitors-A sting in the tale of ENPP1. Molecules 2019;24:1:4192. 10.3390/molecules24224192 PMC6891441 31752288

[45] Flood BA, Higgs EF, Li S, et al. STING pathway agonism as a cancer therapeutic. Immunol Rev 2019;290:24–38. 10.1111/imr.12765 31355488 PMC6814203

[46] Lai J, Mardiana S, House IG, et al. Adoptive cellular therapy with T cells expressing the dendritic cell growth factor Flt3L drives epitope spreading and antitumor immunity. Nat Immunol 2020;21:914–26. 10.1038/s41590-020-0676-7 32424363

[47] Lindeboom RGH, Vermeulen M, Lehner B, et al. The impact of nonsense-mediated mRNA decay on genetic disease, gene editing and cancer immunotherapy. Nat Genet 2019;51:1645–51. 10.1038/s41588-019-0517-5 31659324 PMC6858879

