# Supplementary material for: Epitope spreading driven by the joint action of CART cells and pharmacological STING stimulation counteracts tumor escape via antigen-loss variants
Source: J Immunother Cancer. 2021 Nov 21;9(11):e003351. doi: 10.1136/jitc-2021-003351 (PMC8609946; doi:10.1136/jitc-2021-003351)
Supplement: Supplementary data [file jitc-2021-003351supp001.pdf]

SUPPLEMENTAL METHODS

Mouse strains

7-week-old C57BL/6 mice were obtained from Harlan Laboratories. CD45.1 (B6.SJL-PtprcaPep3b/BoyJ), Rag1-KO (B6.129S7-Rag1tm1Mom/J) and STING-KO (C57BL/6J-Tmem173^{gt}/J) mice were from the Jackson Laboratory. Batf3-KO (B6.129S(C)-Batf3^{tm1Kmm}/J) mice were kindly provided by Dr. K.M. Murphy. All these strains were bred in our animal facility (CIMA, Spain) under specific pathogen-free conditions. Perforin (Perf)-deficient (C57BL/6-Prf1^{tm1Sdz}/J) mice were kindly provided by Dr. H. Hengartner (University Hospital Zurich) and maintained at the Center for Biomedical Research of Aragon (CIBA).

Cell lines and culture medium

Platinum-E (Plat-E) (Cell Biolabs, Inc.) were cultured in Plat-E medium [DMEM-Glutamax (Gibco), FCS 10% (Sigma), sodium pyruvate 1%, essential amino acids 1%, HEPES, 100U/mL penicillin (P), 100 µg/mL streptomycin (S), all from Gibco] supplemented with Puromycin (1µg/ml) and Blasticidin (10 µg/ml). The mouse melanoma B16F10 cell line and B16F10 cells expressing OVA (B16OVA) were verified by Idexx Radil. The B16-PSMA tumor cell line was generously gifted by Dr. F. Pastor (CIMA, Spain). These cell lines were generated by transduction of B16-F10 with amphotropic retrovirus coding a mutated human PSMA version.[1] PSMA is known to undergo constitutive endocytosis via clathrin. The mutated version lacks the MXXXL motif located at the N-terminus of the protein responsible for PSMA internalization[2] and, therefore, exhibits enhanced surface expression. B16-derived cells present the H-2K^b-restricted CD8 T-cell epitope derived from the p15E envelope glycoprotein of murine leukemia virus (MuLV p15E$_{604-611}$, henceforth M8). B16OVA cells also present the H-2K^b-restricted CD8 T-cell epitope derived from ovalbumin (OVA$_{257-264}$). These two epitopes were used to evaluate the epitope spreading effect. Tumor cell lines were cultured in complete medium [RPMI-1640-glutamax, 10% fetal bovine serum, 100 U/mL P, 100 µg/mL S, 10 mg/mL gentamicin, 1 mM HEPES, 50 mM 2-mercaptoetanhol], in an incubator humidifier with 9% CO_2 at 37ºC and were certified as being Mycoplasma-free by using the MycoAlert Mycoplasma Detection Kit (Lonza).

Retrovirus production

Retroviral particles were generated using Plat-E cell-mediated transfection. Plat-E cells (7×10^5 cells/well) were seeded 24h before transfection in 6-well plates in 2 mL/well of infection medium (Plat-E medium without P/S). 20-24h later, once the cells had reached 70% confluence, 500µL/well of

a mix containing plasmids and Lipofectamine 2000 (Thermo Fisher Scientific) were added. The mixtures contained 3 µg/well of transgene carrying plasmid, 2 µg/well of pCL-Eco (helper plasmid) and 10 µL/well of Lipofectamine 2000, and were prepared in OPTIMEM medium according to the manufacturer´s protocol. pCL-Eco was a gift from Inder Verma[3] (Addgene plasmid #12371). Plat-E medium was changed 24h post-transfection. The supernatant containing the retroviruses was collected 48h and 72h post-transfection. Debris was removed by centrifugation at 2000rpm for 1 minute. Supernatants were kept at 4ºC until T-cell transduction.

Retroviral transduction of mouse T cells

Isolated CD4 and CD8 T cells (10^6/well) were separately activated in 24-well plates (Cellstar) coated with anti-CD3 (145-2C11) (2 µg/ml) and soluble anti-CD28 (37.51) (1 µg/ml) (both mAb from Biolegend) in complete medium containing human IL-2 (50 U/ml) (Proleukin) at 10^6/mL density. After 48h of activation, cells were resuspended in retrovirus containing supernatant supplemented with protamine sulfate (10 µg/mL, SIGMA) and human IL-2 (50 IU/mL) and 'spin-inoculated' at 2000g for 90 min at 32 ºC. This last process was repeated with fresh retrovirus supernatant the next day. Transduction efficiency was evaluated 2 days after the first injection by measuring reporter protein (EGFP) expression by flow cytometry.

CAR surface staining

Four to five days after retroviral transduction, T cells were first incubated (45', room temperature) with a biotinylated goat F(ab')$_2$ fragment polyclonal antibody specific for mouse IgG (H+L) (Jackson Immunoresearch). This Ab recognizes the murine scFV of CAR-gp75 and CAR-PSMA, but not that of CAR-CD19. As a control, cells were incubated with biotinylated goat F(ab')$_2$ fragment polyclonal antibody specific for rat IgG (H+L) (Jackson Immunoresearch). Then, cells were stained with Streptavidin-APC (allophycocyanin) (BD Biosciences) (15', 4ºC) and analyzed by flow cytometry

Tissue and blood processing

Tumor-bearing mice were sacrificed and excised tumors were incubated in a dissociation solution (RPMI-1640-glutamax supplemented with 400U/mL D collagenase (Roche) and 50 µg/mL of DNase I (Roche) for 30 minutes at 37ºC. Tumors were mechanically disaggregated, filtered through a 70µm nylon cell strainer (FALCON) and centrifuged at 2000rpm for 8 minutes. After erythrocyte lysis with ACK buffer (NH_4Cl 0.15M, $KHCO_3$, 0.01M and Na_2EDTA 0.1mM), cells were resuspended in appropriate medium and counted. DLN and CLN were homogenized by mechanical dissociation through a 70µm nylon cell strainer and treated with ACK buffer to remove erythrocytes. Blood (100

μl) was collected in tubes containing 5 μl of Sodium Heparin 5% (Hospira) and lysed with Red Blood Cell (RBC) buffer (Biolegend).

Recognition of cognate tumor cells by CARTs

The cytotoxic activity of CAR-PSMA T cells against B16-PSMA cells was measured using real-time killing assays and the 7-aminoactinomycin D (7-AAD) cell-mediated cytotoxicity assay. Real-time killing assays were performed by measuring electric impedance over time in an xCELLigence Real Time Cell Analysis Instrument (ACEA). Briefly, B16-PSMA or B16OVA (negative control) cells (5×10^4 cells/well) were grown on xCELLigence E-plates (Agilent). At 24h, medium or WT CAR CD8 T cells [CAR-CD19 or CAR-PSMA] (1×10^5 cells/well) were added. Electric impedance was measured every 5 min for 24h. Then, the supernatant from E-plates was recovered and IFNɣ levels were measured by ELISA [Mouse IFNɣ BD OptEIA Set (BD Bioscience)] according to the manufacturer's instruction. For the 7AAD cell-mediated cytotoxicity assays, WT or Perf-KO (CD8 or CD4) T cells expressing CAR-PSMA or CAR-CD19 (1×10^5 cells/well) were co-cultured (12h) with B16-PSMA or B16OVA tumor cells (5×10^4 cells/well) (test wells) in 96-well culture plates. As a control, tumor cells were cultured alone (control wells). Then, cells were stained with anti-CD45 (30F11, Biolegend) mAb and 7AAD. The percentage of dead tumor cells ($7AAD^+CD45^-$) from total tumor cells ($CD45^-$) was analyzed by flow cytometry. The percentage of specific lysis was calculated as: ([%$7AAD^+$ target test well]-[mean %$7AAD^+$ targets control well])/(100-[mean %$7AAD^+$ target control well]) * 100.

To assess the ability of CAR-gp75 T cells to recognize the gp75 Ag, CD8 CARTs were co-cultured with a cell suspension obtained from implanted B16OVA or MC38 (negative control) tumors. Briefly, one week after tumor inoculation, mice were sacrificed and excised tumors were processed as described above. Cells were resuspended in complete medium and counted. Different quantities of tumor cells ranging from 0 to 2×10^5 cells were co-cultured with 1×10^5 CARTs for 24h. IFNɣ levels were measured in the supernatants by ELISA.

PSMA and gp75 staining in tumor cells

For PSMA surface staining of *in vivo* growing tumor cells, tumor-bearing mice were sacrificed and their tumors homogenized to single-cell suspension as described above. Tumor-cell suspensions were stained with Zombie NIR (ZN) Fixable dye (Biolegend) and then with a mix containing fluorochrome-labeled anti-hPSMA (LNI-17) and -CD45 (30F11) mAbs and purified anti-CD16/32 (93, Fc Block) mAb. As a control, cells were stained in the same way but with the isotype control corresponding to anti-PSMA (mIgG1) mAb. For gp75 surface staining, tumor-cell suspensions were stained with anti-gp75 (TA99, IgG2a) mAb or purified mIgG2 (control isotype) together with fluorochrome-labeled

anti-CD45 (30F11) and Fc Block. Then, cells were stained with a fluorochrome-labeled anti-mouse IgG2a (RMG2a-62). Surface expression of hPSMA or gp75 was determined by flow cytometry in tumor cells ($CD45^{-}FSC^{hi}SSC^{hi}$). For gp75 total (surface plus intracellular) staining, cells were first stained with ZN and anti-CD45 mAb, then permeabilized and fixed (Cytofix/cytoperm, BD Biosciences), stained with purified anti-gp75 mAb or control isotype and finally stained with fluorochrome-labeled anti-mouse IgG2a.

For PSMA and gp75 surface staining of *in vitro* cultured tumor cells, cells were stained as described for the *ex vivo* characterization of *in vivo* growing tumor cells but without anti-CD45 mAb. In some experiments, tumor cells were cultured *in vitro* with or without mouse IFNγ (100 UI/ml) (Inmunotool) for 48h and then they were surface stained for gp75 detection. All mAb were from Biolegend except for anti-gp75 (TA99) (bioXcell). Cells were acquired in a FACSCanto-II (BD Biosciences) or a CytoFlex (Beckman Coulter) flow cytometer. Data were analyzed with FlowJo software (Tree Star).

Cell staining for *ex vivo* characterization of CART and endogenous T cells

Tissue-cell suspensions and blood cells were incubated with ZN Fixable dye (Biolegend). Subsequently, cells were stained with phycoerythrin-labeled H-2Kb/OVA257-264-tetramer (Tetramer-shop) and APC-labeled H-2Kb/M8 (MuLV p15E604-611)-tetramer (MBL) and, after washing, with fluorochrome-conjugated mAbs against mouse CD8 (KT15), CD4 (RM4-5), CXCR3 (Cxcr3-173), CD25 (PC61-5), CD137 (17B51H1), PD-1 (29F-1A12), CD45 (30F11) and CD45.1 (A20) in the presence of Fc-Block [anti-CD16/32 (93)]. Then, cells were fixed and permeabilized with Cytofix/CytoPerm (BD Biosciences) and intracellular stained with a fluorochrome-conjugated anti-human/mouse Granzyme B (QA16A02) mAb in the presence of Fc-Block. All mAb were from Biolegend except for anti-CD8 (KT15) (Thermofisher). Cells were acquired in a FACSCanto-II (BD Biosciences) or a CytoFlex (Beckman Coulter) flow cytometer. The absolute numbers of cells was determined by flow cytometry using a volumetric cytometer (Cytoflex). Data were analyzed with FlowJo software (Tree Star)

In-depth analysis of the TCR repertoire

Approximately 100 μl of blood were collected in tubes containing 10 ul of 50 mM EDTA. Erythrocytes were lysed with 500 μl of RBC (Biolegend). Tumors were homogenized to single-cell suspensions as described above. After washing in PBS, blood cells and tumor-cell suspensions were pelleted and frozen at -80ºC. Genomic DNA (gDNA) was isolated using the NucleoSpin Tissue Kit (Macherey-Nagel) reaching a DNA Integrity Number score >8. Five-hundred ng (unless otherwise indicated; supplemental Table 1) or 1500 ng of gDNA from blood cells or tumor-cell suspensions, respectively,

were used for TCR sequencing with the Oncomine™ mouse TCR Beta-SR DNA Assay (Thermo Fisher Scientific) according to the manufacturer's instructions. Library construction and sequencing (IonTorrent S5) was performed in the Genomics Facility at CIMA Lab Diagnostics (Spain, Pamplona). Individual clonotypes were identified and bioinformatics analysis of the data was performed with the help of the IonReporter software. Using CDR3 counts, the measures of diversity indices for each sample were calculated using the PAST (version 4.03) (PAlaeontological STatistics) data analysis package.[4] The Gini coefficient was calculated using the Gini coefficient calculator developed by Dr. B. Shlegeris (https://shlegeris.com/gini.html).

Statistical analysis

Statistical tests were performed using GraphPad Prism (v.8.4.0). Tumor growth curves were compared using nonlinear regression (curve fit). Differential survival was assessed using the Log-rank (Mantel-Cox) test. For simple comparisons, unpaired (non-parametric Mann-Whitney test, two-tailed) and paired (Wilcoxon matched-pairs signed rank tests) two-tailed Student's t tests were used. Multiple comparisons were performed using One-way ANOVA and the nonparametric Kruskal-Wallis test followed by Dunns post-test. Significance was set at $p < 0.05$.

REFERENCES

1 Pastor F, Kolonias D, McNamara JO, *et al.* Targeting 4-1BB costimulation to disseminated tumor lesions with Bi-specific oligonucleotide aptamers. *Mol Ther* 2011;**19**:1878–86. doi:10.1038/mt.2011.145

2 Rajasekaran SA, Anilkumar G, Oshima E, *et al.* A Novel Cytoplasmic Tail MXXXL Motif Mediates the Internalization of Prostate-specific Membrane Antigen. *Mol Biol Cell* 2003;**14**:4835–45. doi:10.1091/mbc.E02-11-0731

3 Naviaux RK, Costanzi E, Haas M, *et al.* The pCL vector system: rapid production of helper-free, high-titer, recombinant retroviruses. *J Virol* 1996;**70**:5701–5. doi:10.1128/jvi.70.8.5701-5705.1996

4 Hammer Ø, Harper DAT, Ryan PD. Past: Paleontological statistics software package for education and data analysis. *Palaeontol Electron* 2001;**4**:178.

SUPPLEMENTARY FIGURES

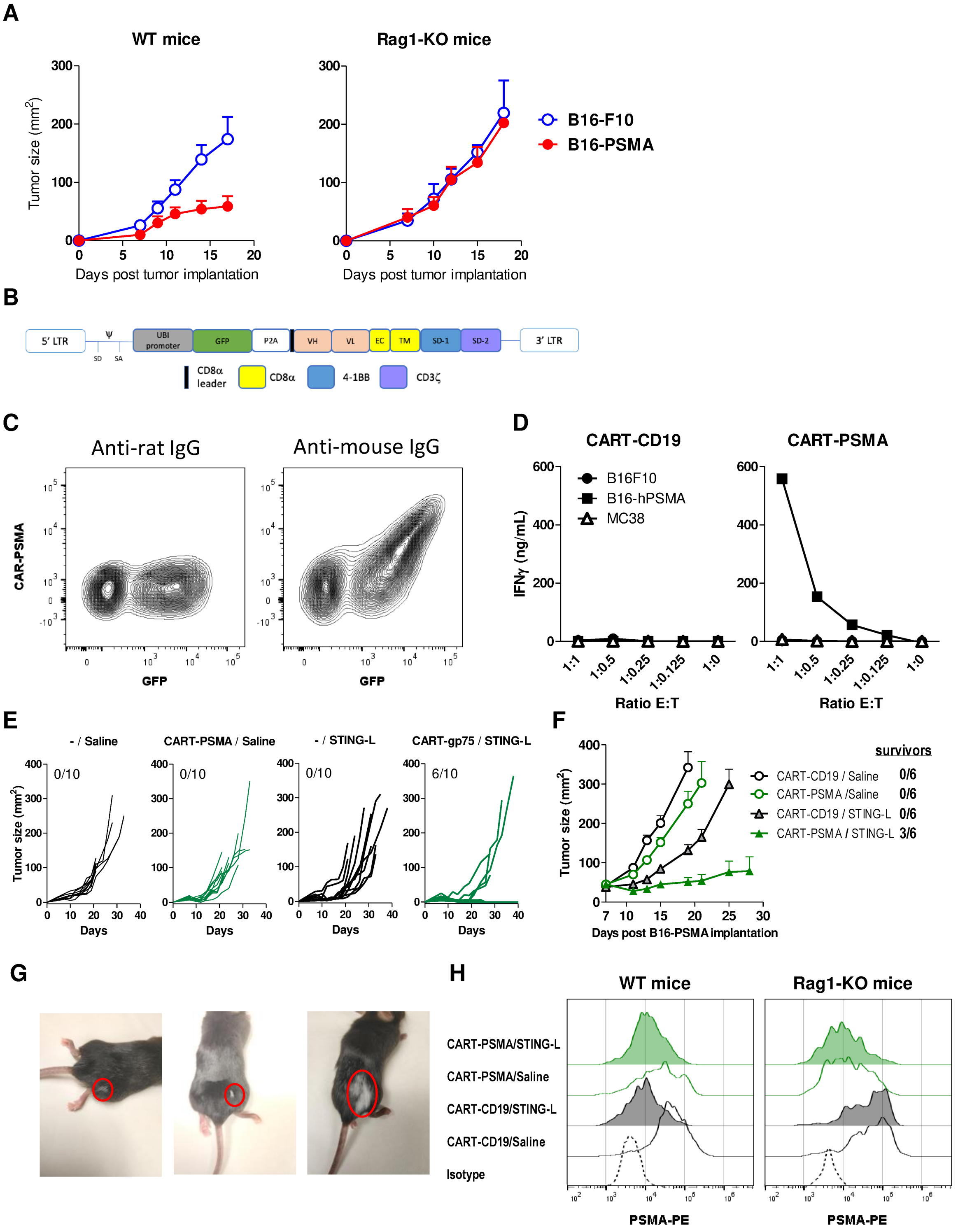

Figure S1. Defining the B16-PSMA/CAR-PSMA tumor model. (A) B16-PSMA and B16-F10 tumor growth kinetics *in vivo*. WT or Rag1-KO mice (n= 8 mice/group) were injected subcutaneously with 5×10^5 B16-PSMA or B16-F10 tumor

cells and tumor growth was followed. (B) Illustration of the retroviral plasmid bearing the *Car-psma* gene. This synthetic gene is flanked by two LTR sequences (Long Terminal Repeat) necessary for the integration into the host genome. The gene is under the constitutive expression of the ubiquitin (UBI) promoter, along with EGFP used to determine transduction efficiency. The P2A sequence works as a spacer between EGFP and CAR-PSMA and is cleaved automatically when translated into peptide. The PSMA binding region contains the VH (Variable Heavy) y VL (Variable Light) of J591 mouse hybridoma joined by a linker. This extracellular domain is followed by the extracellular (EC) and transmembrane domains (TM) of CD8α, and by an intracellular region containing the signaling domains of 4-1BB and CD3ζ. (C) Detection of CAR surface expression on murine T cells transduced with CAR-PSMA retroviral vector. ScFv was detected using an antibody against mouse IgG. An anti-rat IgG antibody was used as a negative control. (D) *In vitro* recognition of B16-hPSMA by CAR-PSMA T cells. B16-hPSMA, B16F10, or MC38 cells (the last two used as control) were cultured (24h) with CART-PSMA or CART-CD19 CD8 cells (in triplicate) at different CART cell:tumor cell ratios. IFNγ levels were measured in the culture supernatant by ELISA. (E) Tumor growth kinetics in individual mice from the experiment shown in figure 2B. (F) 7-day B16-PSMA s.c. tumor-bearing B6 mice received an i.v. dose (3×10^6) of CART-PSMA or CART-CD19 cells. 2’3’cGAMP (5 µg) or saline was injected intratumorally (i.t.) on days 11 and 17 of tumor implantation. Data represent mean tumor size progression. (E and F) Ratio of survivors/total mice is shown. (G) Pictures of three mice treated with the CART-PSMA/STING-L combination (fig. 2B) showing areas affected by vitiligo. (H) Histograms showings the Median Fluorescence Intensity of hPSMA or control isotype in tumor cells from representative mice from the experiment shown in figure 2D. Data are represented as mean±SEM (A and F) and mean±SD (D). One experiment representative of 2 (A, F, G and H) or at least 3 (C and D) experiments.

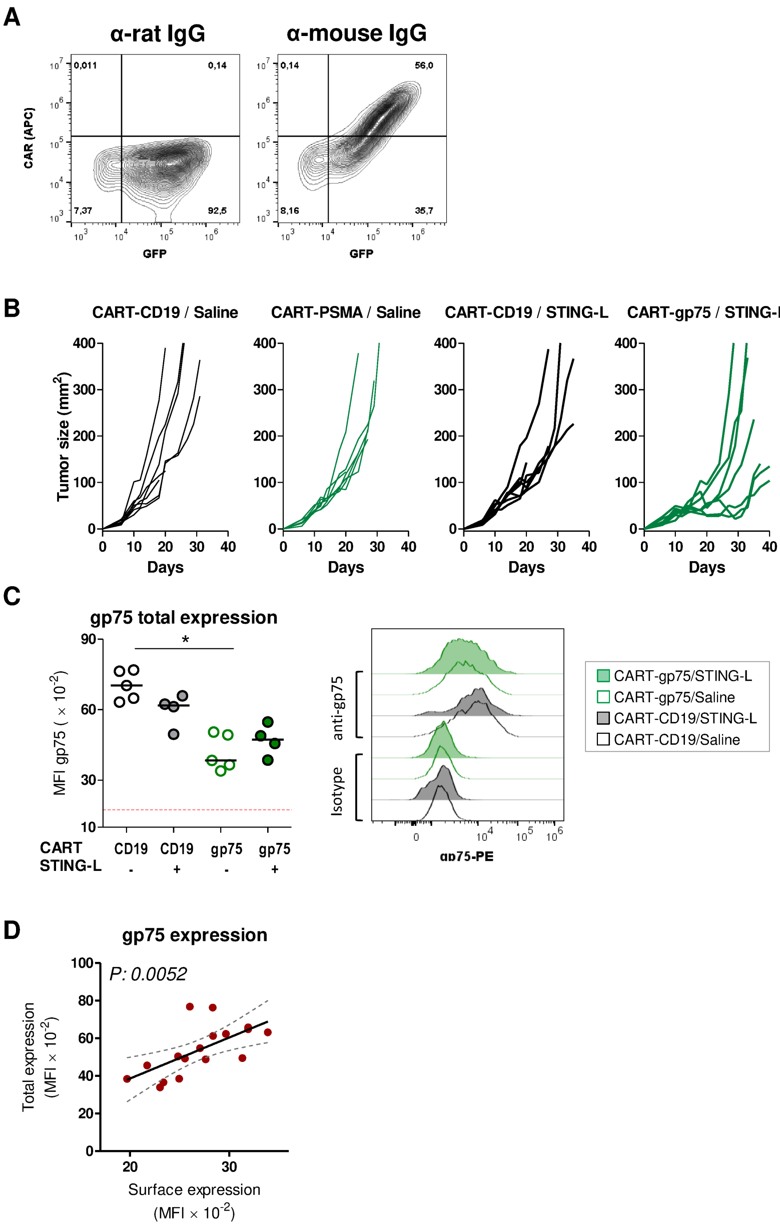

Figure S2. Defining the B16OVA/CAR-gp75 tumor model. (A) Detection of CAR surface expression on murine T cells transduced with CAR-gp75 retroviral vector. ScFv was detected using an antibody against mouse IgG. An anti-rat IgG antibody was used as a negative control. (B) Tumor growth kinetics in individual mice from the experiment shown in figure 2E. (C) CART-cell editing of gp75 expression. Mice treated as in figure 2F, were sacrificed on day 20 and their tumors were homogenized to a single cell suspension. Total expression of gp75 was determined by flow cytometry in tumor ($CD45^-$ $FSC^{hi}SSC^{hi}$) cells. Red dotted lines in box&whiskers graphs (left) depict the average Median Fluorescence Intensity (MFI) of tumor-cell staining with the IgG isotype. Histograms on the right show the MFI of gp75 or control isotype in tumor cells from a representative mouse in each group. (D) Graph showing the directly proportional relationship between the surface expression and the total expression of gp75. Data are represented as mean±SEM (B). One-way ANOVA and nonparametric Kruskal-Wallis test and Dunn's post-test to compare all pairs of groups (C). Linear regression test (D).

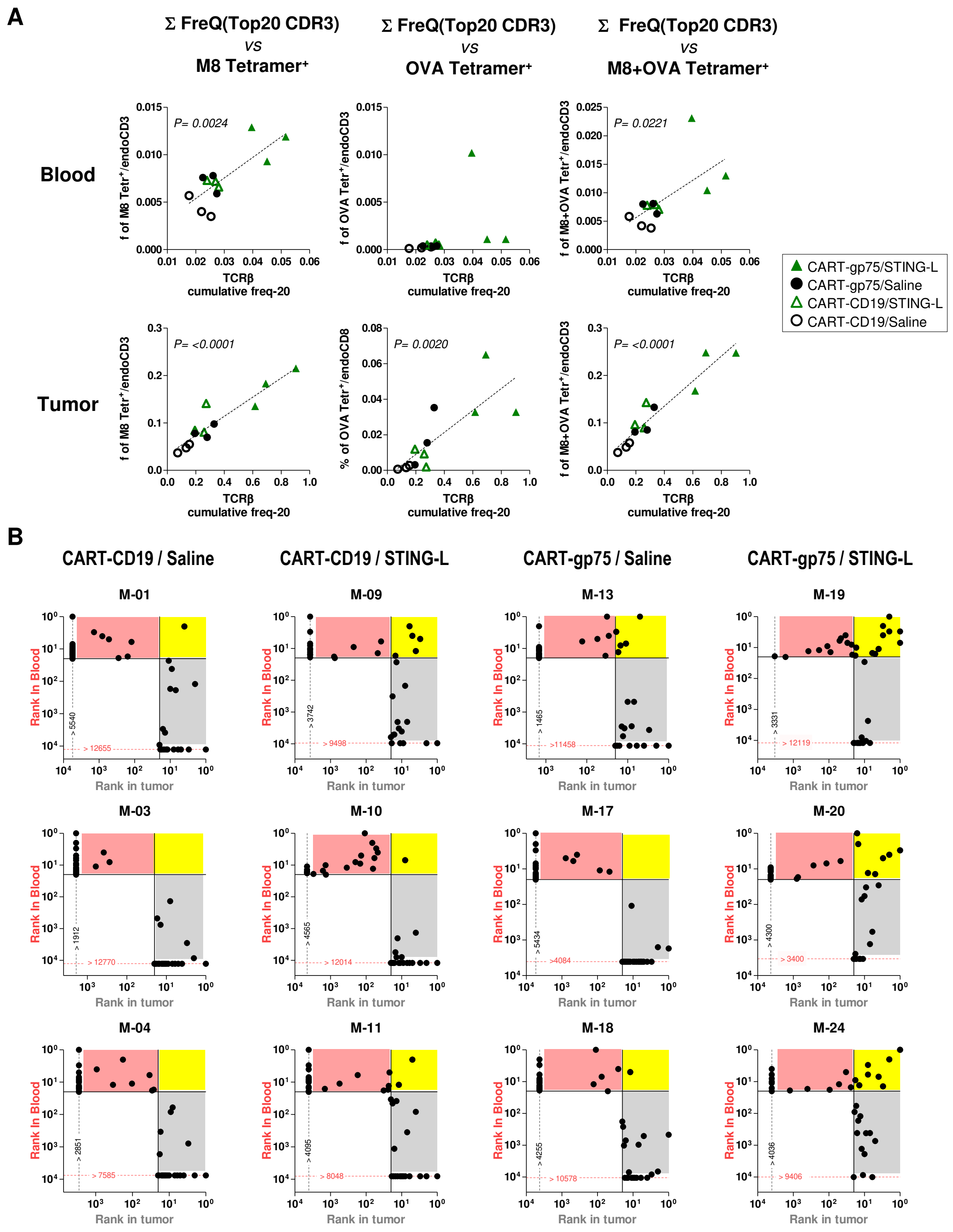

Figure S3. Analysis of the endogenous TCR repertoire upon CART +/- STING-L treatment. (A) Directly proportional relationship between the cumulative frequency of the TOP20 clones and the percentage of Tetramer+ cells in blood and treated tumors. Mice were treated as in Figure 4. The percentage of M8 and OVA Tetramer+ cells within endogenous CD3 T cell subsets in blood and saline/STING-L treated tumor was determined by flow cytometry. gDNA were isolated from peripheral blood cells of 3 mice representative of each group and used for bulk CDR3 TCRβ sequencing. The cumulative

frequency of the TOP20 clones was plotted against the percentage of M8 (left) and OVA (middle) tetramer⁺ cells and the sum of the percentages of each of these tetramer⁺ populations (M8+OVA) (right). Each point represents a mouse. Linear regression test. Since the percentages of OVA tetramer⁺ cells were very low in the blood of unresponsive groups, the correlation with OVA tetramer⁺ cells was not studied. (B) CDR3β matching between blood and tumor TOP20 clones. The tumor and blood TOP20 clonotypes are plotted according to their rank in the tumor (x axis) and blood (y axis) TCR repertoire. Vertical and horizontal dotted lines show the CDR3β detection threshold in tumor and blood, respectively. The CDR3β detection threshold was defined as the highest clonotype rank detected in tumor (gray dotted line) or blood (red dotted line). Those clonotypes that were not detected in tumor or blood were considered to occupy a rank higher than that defined by the threshold and were plotted in the threshold line. The yellow area shows those CDR3β shared by blood and tumor that are among the TOP20 in both tissues. The gray and pink areas show the shared clonotypes that are among the TOP20 in tumor or in blood, respectively.

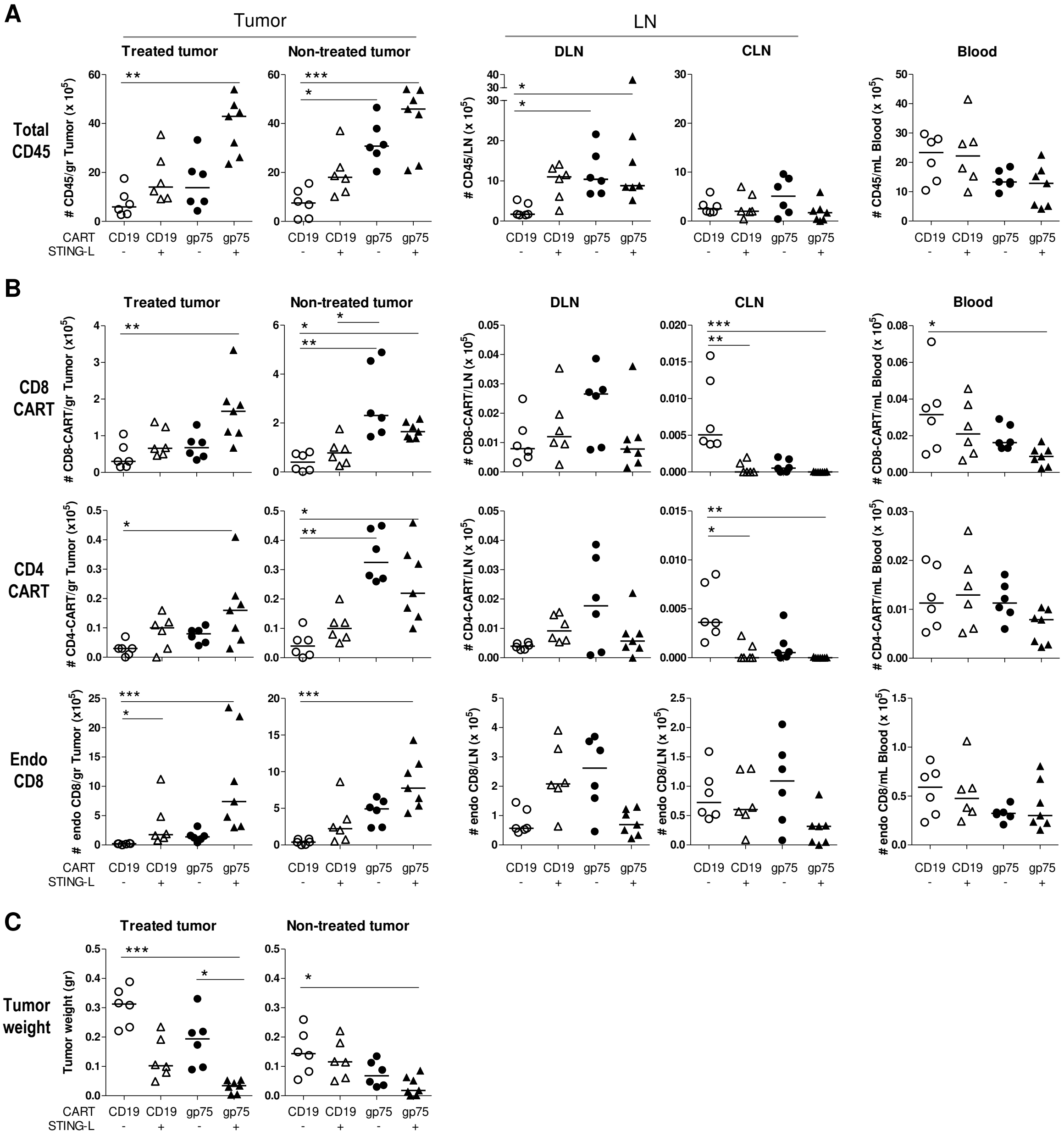

Figure S4. CART/STING-L combination enhances the infiltration of CART and endogenous T cells in STING-L-treated and -non-treated tumors. B16OVA-tumor bearing C57BL6 ($CD45.2^+$) mice were treated with STING-L and CART cells as in figure 4. CART cells were generated in CD45.1 mice. On day 17 they were bled and sacrificed. Cell suspensions obtained from STING-L-treated and –non-treated tumors, lymph nodes (LN) [both, DLN and CLN (relative to the STING-L-treated tumor)] and blood were stained and analyzed by flow-cytometry. CD8 and CD4 CART cells were identified as $GFP^+CD45.1^+CD8^+$ and $GFP^+CD45.1^+CD8^-$ cells, respectively. Endo-CD8 cells were identified as $GFP^-CD45.1^-CD8^+$ cells. (A and B) Number (#) of $CD45^+$ cells [including both endogenous ($CD45.2^+$) and CART cells ($CD45.1^+$)] (A) and that of CD8 CART, CD4 CART and endo-CD8 cells (B) expressed as cell number/per grame (gr) of tumor (in tumors), total cell counts per LN (in DLN and CLN) and cells/ml of blood (in Blood). The absolute cell numbers were determined by flow cytometry using a volumetric cytometer (Cytoflex). (C) Weight of tumors collected after sacrifice. (A-C) Each point represents a mouse and medians are plotted. One-way ANOVA and post-test to compare all pairs of groups. Only comparisons with statistical significance are given. ***p < 0.0005, **p < 0.005, *p < 0.05. One experiment representative of two is shown.

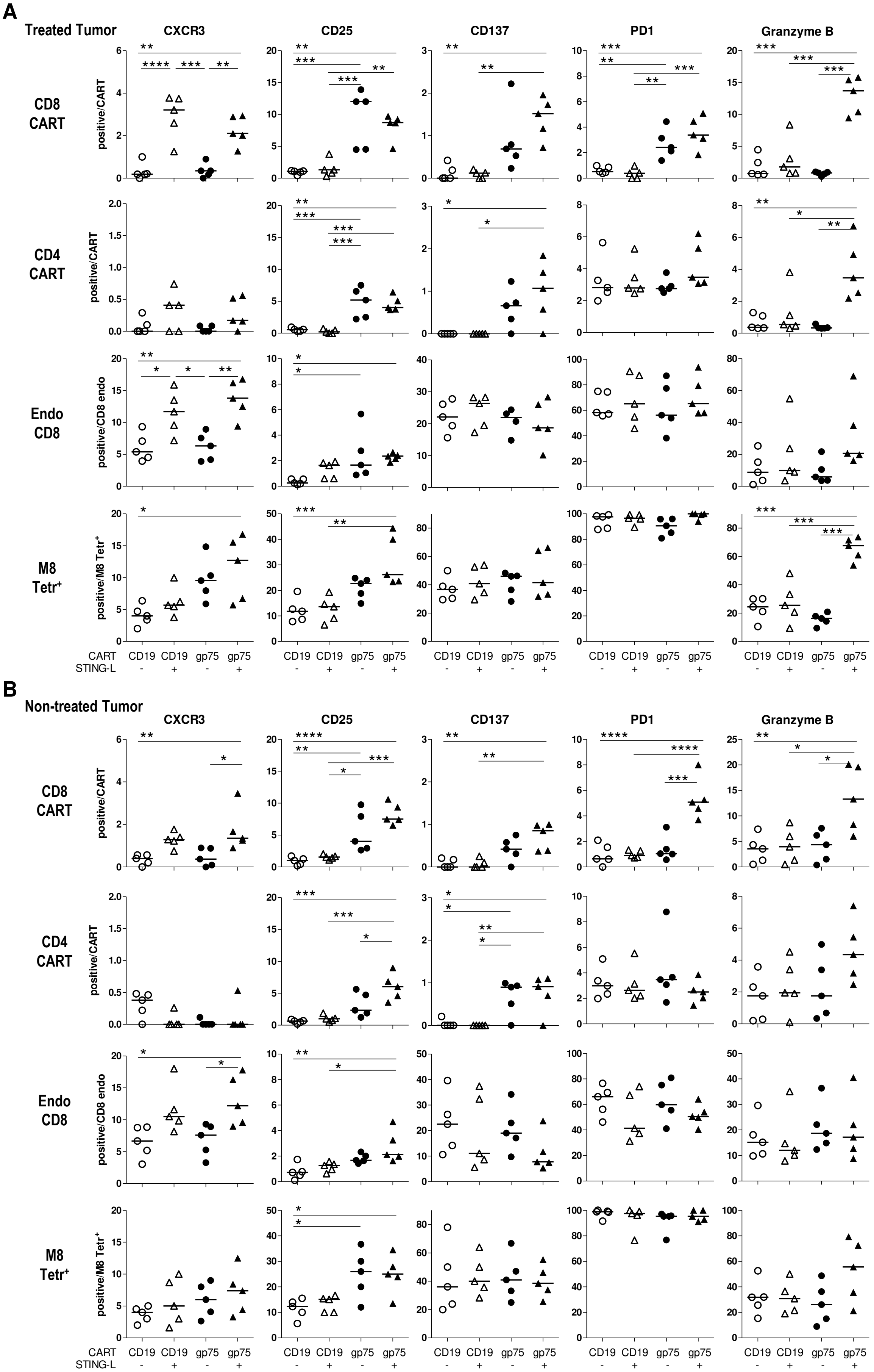
A
Treated Tumor
CXCR3
CD25
CD137
PD1
Granzyme B
CD8 CART
CD4 CART
Endo CD8
M8 Tetr+
positive/CART
positive/CD8 endo
positive/M8 Tetr+
CART
STING-L
CD19
gp75
B
Non-treated Tumor

Figure S5. CART/STING-L combination enhances the activation of CART and endogenous T cells in STING-L-treated and -non-treated tumors. (A and B) B16OVA-tumor bearing C57BL6 ($CD45.2^+$) mice were treated with STING-L and CART cells as in figure 4. CART cells were generated in CD45.1 mice. On day 17 they were bled and sacrificed. Cell suspensions obtained from STING-L-treated (A) and –non-treated tumors (B) were stained and analyzed by flow-cytometry. CD8 and CD4 CART cells were identified as $GFP^+CD45.1^+CD8^+$ and $GFP^+CD45.1^+CD8^-$ cells, respectively. Endo-CD8 cells were identified as $GFP^-CD45.1^-CD8^+$ cells. M8 Tetr cells were identified as $GFP^-CD45.1^-CD8^+M8Tetr^+$ cells. Each point represents a mouse and the median is given. One-way ANOVA and post-test to compare all pairs of groups. Only comparisons with statistical significance are indicated. ****$p < 0.0001$, ***$p < 0.0005$, **$p < 0.005$, *$p < 0.05$. One experiment representative of two is shown.

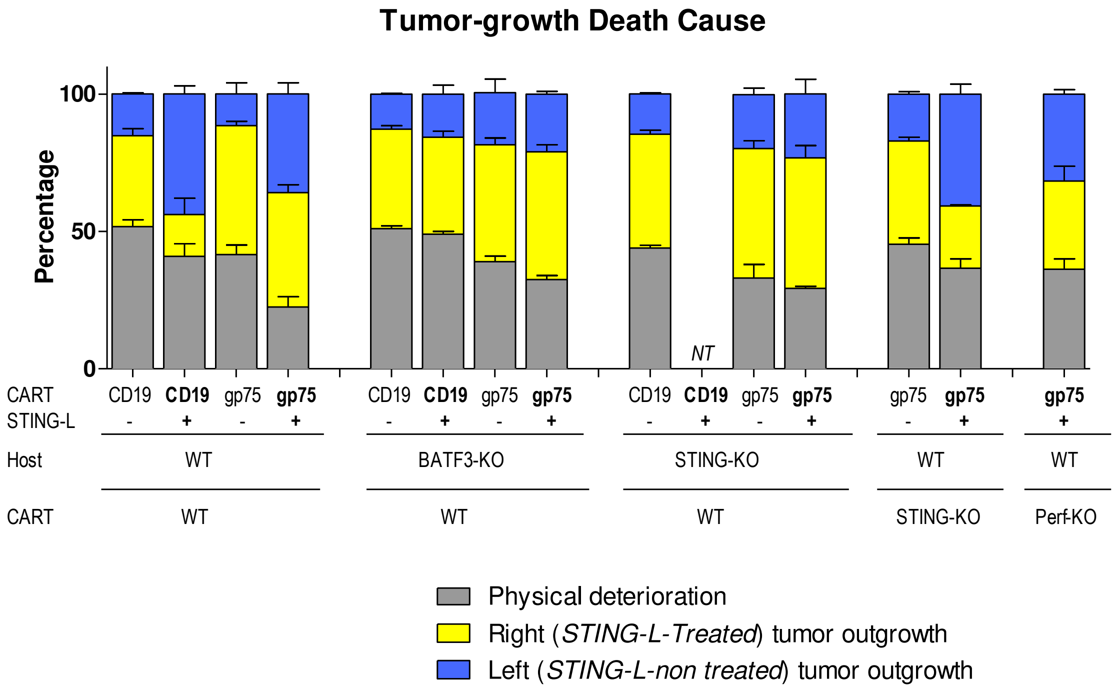

Figure S6. Tumor-growth death cause. The death cause of B16OVA tumor-bearing mice treated with CART/STING-L therapy was analyzed through all the experiments done. Death was considered to be due to outgrowth of the right tumor or outgrowth of the left tumor if the mean diameter of the right or left tumor, respectively, reached 20 mm. Death was considered to be due to physical impairment if the mouse had to be euthanized by applying one or more of the following criteria: impaired mobility, signs of lethargy and lack of physical activity, weight loss and/or ulcerated/necrotic tumor from more than 72 hours. The graph shows the data of (from left to right): (i) CART/STING-L therapy in tumor-bearing WT mice treated with CART cells derived from WT mice (WT CART) (mean of 8 experiments). (ii) CART/STING-L therapy in tumor-bearing Batf3-KO mice treated with WT CART cells (mean of 2 experiments). (iii) CART/STING-L therapy in tumor-bearing STING-KO mice treated with WT CART cells (mean of 2 experiments) [the CART-CD19/STING-L treatment was non tested (*NT*) in this setting]. (iv) CART+/-STING-L therapy in tumor-bearing WT mice treated with STING-ko CART cells (mean of 2 experiments); (v) CART+STING-L therapy in tumor-bearing WT mice treated with Perf-ko CART cells (mean of 2 experiments). Only those groups that were tested are exhibited (iv and v).

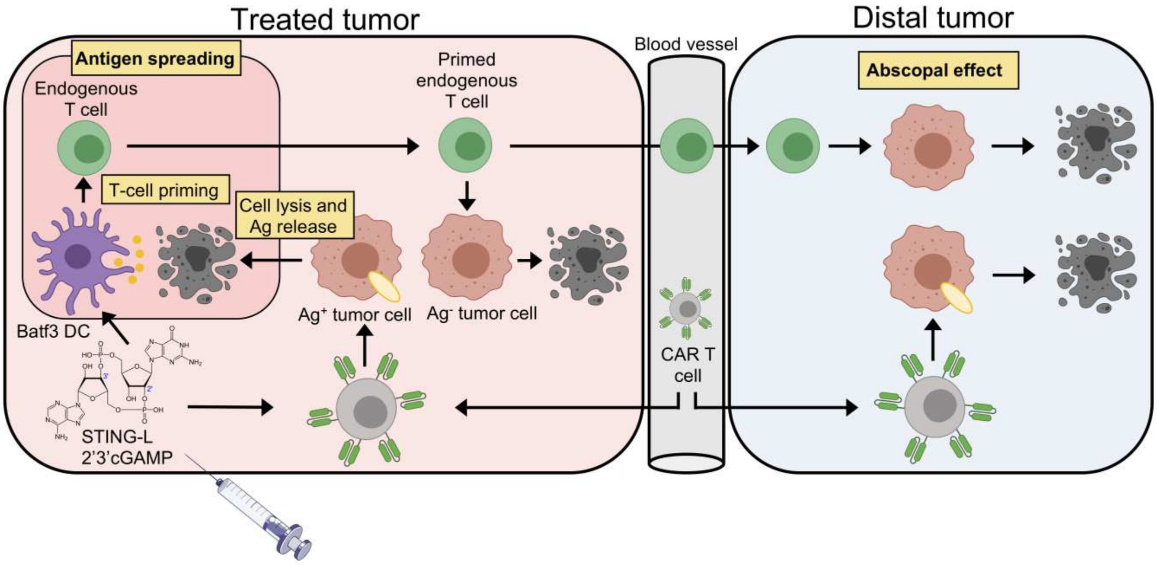

Figure S7. Graphical summary

- CART cells drive the selection of tumor cells expressing low target antigen levels.
- CART cells and intratumoral administration of 2'3'cGAMP enhance antitumor activity and exert abscopal effects in distal non-STING-L-treated tumors.
- CART/STING-L combination fosters a local and systemic endogenous T-cell response against non-CAR-targeted tumor antigens.
- CART/STING-L combination enhances the infiltration and activation of CART and endogenous T cells in STING-L-treated and -non-treated tumors.
- The synergistic effect of the combination relies on host STING signaling and Batf3-dependent DCs, and partially depends on perforin release by CART cells.

Supplementary Table 1. Definition and range of values of the indices used to measure diversity in the TCRβ repertoire

	Range and meaning		
**Diversity Index**	0	1	High value
The Gini Coefficient measures the inequality among values of a frequency distribution.	diversity	clonality	///
Dominance (D) measures the degree to which a CDR3 clonotype is more numerous than its competitors in a TCR repertoire.	diversity	clonality	///
The Berger-Parker index measures the numerical importance of the most abundant species.	diversity	clonality	///
The Simpson (1-D) measures 'evenness' of the TCR repertoire. It ranges between 0 (frequency dominated by one clone, repertoire clonality) and 1 (all clones are equally frequent – repertoire diversity).	clonality	diversity	///
The Shannon index (H) takes into account the number of individuals (counts) as well as number of taxa (CDR3). It is more sensitive to differences in sample size (counts) than the Simpson index.	clonality		diversity
Evenness quantifies how equal the TCR repertoire is numerically.	clonality	diversity	///
Equitability (J) is the Shannon diversity divided by the logarithm of the number of CDR3 clonotypes.	clonality	diversity	///

Supplementary Table 2. Diversity analysis of the TCRβ repertoire in blood

group	(1) CART-CD19 / Saline			(2) CART-CD19 / STING-L			(3) CART-gp75 / Saline			(4) CART-gp75 / STING-L			ANOVA (§)
sample / **PARAMETER (&)**	**S-1**	**S-2**	**S-3**	**S-4**	**S-5**	**S-6**	**S-7**	**S-8**	**S-9**	**S-10**	**S-11**	**S-12**	
Taxa_S (# CDR3)	1,28E+04	1,29E+04	7,67E+03	9,59E+03	1,21E+04	8,11E+03	1,16E+04	4,88E+03	1,07E+04	1,22E+04	3,42E+03	9,46E+03	
Individuals (counts)	1,45E+06	2,22E+06	1,53E+06	8,49E+05	9,64E+05	1,51E+06	1,17E+06	1,64E+06	1,25E+06	1,24E+06	1,80E+06	1,27E+06	
**Gini coefficient**	0.506	0.521	0.491	0.513	0.459	0.514	0.497	0.531	0.55	0.509	0.54	0.535	*0.3685*
**Dominance_D**	0.0001807	0.0001732	0.0002689	0.0003061	0.0001962	0.000262	0.0002132	0.0004184	0.0002278	0.000345	0.0007194	0.0003941	*0.118*
**Berger-Parker**	0.003264	0.003114	0.003244	0.005344	0.005589	0.002765	0.004273	0.002027	0.002602	0.01017	0.00867	0.01105	***0.0572***
**Simpson_1-D**	0.9998	0.9998	0.9997	0.9997	0.9998	0.9997	0.9998	0.9996	0.9998	0.9997	0.9993	0.9996	*0.2097*
**Shannon_H**	9.005	8.995	8.522	8.573	9.021	8.543	8.915	8.008	8.75	8.903	7.619	8.617	*0.7641*
**Evenness_e^H/S**	0.6362	0.6246	0.6552	0.5516	0.6825	0.6326	0.6431	0.6152	0.5915	0.601	0.5952	0.5837	*0.3395*
**Equitability_J**	0.9522	0.9503	0.9527	0.9351	0.9594	0.9491	0.9528	0.9428	0.9434	0.9459	0.9362	0.9412	*0.3466*
**ng gDNA (#)**	500	500	210	500	500	206	500	136	500	500	81	500	

(§) Kruskal-Wallis Test. P value.

(&) Taxa (S) is defined as the number of productive unique TCRβ CDR3 (identified by the CDR3 nucleotide sequence). Individuals represent the total counts. Diversity indices were calculated as described in the Methods. The meaning of each Diversity index is defined in supplementary Table 1.

(#) 500 ng of blood cell DNA were used for TCR sequencing, except for samples S-3, S-6, S-8 and S-11 where it was not possible to reach this amount and all the gDNA obtained was used for sequencing.

Supplementary Table 3. Diversity analysis of the TCRβ repertoire in treated tumor

group	(1) CART-CD19 / Saline			(2) CART-CD19 / STING-L			(3) CART-gp75 / Saline			(4) CART-gp75 / STING-L			ANOVA (§)	Significant comparison (#)
sample / **PARAMETER (&)**	S-1	S-2	S-3	S-4	S-5	S-6	S-7	S-8	S-9	S-10	S-11	S-12		
Taxa_S (# CDR3)	5,59E+03	1,92E+03	2,88E+03	3,78E+03	4,60E+03	4,14E+03	1,47E+03	5,49E+03	4,30E+03	3,41E+03	4,34E+03	4,08E+03		
Individuals (counts)	1,72E+06	1,74E+06	2,10E+06	1,72E+06	1,14E+06	1,91E+06	1,63E+06	1,61E+06	1,19E+06	1,43E+06	2,21E+06	1,23E+06		
**Gini coefficient**	0.612	0.629	0.666	0.665	0.723	0.699	0.687	0.666	0.688	0.964	0.858	0.869	***0.0367***	(* 1 vs 4)
**Dominance_D**	0.0007221	0.002816	0.002438	0.003211	0.006497	0.01146	0.01219	0.004066	0.01289	0.3188	0.0885	0.1014	***0.0216***	(* 1 vs 4)
**Berger-Parker**	0.007112	0.03523	0.02546	0.02609	0.04931	0.08443	0.07913	0.03853	0.09477	0.5219	0.2458	0.2858	***0.0307***	(* 1 vs 4)
**Simpson_1-D**	0.9993	0.9972	0.9976	0.9968	0.9935	0.9885	0.9878	0.9959	0.9871	0.6812	0.9115	0.8986	***0.0216***	(* 1 vs 4)
**Shannon_H**	7.898	6.771	7.015	7.148	6.898	6.821	6.01	7.397	6.835	2.269	4.752	4.397	*0.0922*	
**Evenness_e^H/S**	0.4813	0.4533	0.3863	0.3368	0.2152	0.2214	0.2768	0.297	0.2163	0.002837	0.0267	0.01993	***0.0249***	(* 1 vs 4)
**Equitability_J**	0.9153	0.8954	0.8806	0.8679	0.8179	0.819	0.8239	0.859	0.817	0.279	0.5674	0.529	***0.0249***	(* 1 vs 4)
**ng gDNA**	1500	1500	1500	1500	1500	1500	1500	1500	1500	1500	1500	1500		

(§) Kruskal-Wallis Test. P value.

(#) Dunn's Multiple comparison tests. The column shows the groups that had significant differences.

(&) Taxa (S) is defined as the number of productive unique TCRβ CDR3 (identified by the CDR3 nucleotide sequence). Individuals represent the total counts. Diversity indices were calculated as described in the Methods. Diversity indices are defined in supplementary Table 1.
